# Discovery of an Interlocked and Interwoven Molecular Topology in Nanocarbons via Dynamic C–C Bond Formation

**DOI:** 10.1021/jacs.5c04268

**Published:** 2025-05-23

**Authors:** Harrison M. Bergman, Angela T. Fan, Christopher G. Jones, August J. Rothenberger, Kunal K. Jha, Rex C. Handford, Hosea M. Nelson, Yi Liu, T. Don Tilley

**Affiliations:** † Department of Chemistry, 1438 University of California, Berkeley, California 94720, United States; ‡ Department of Chemistry and Biochemistry, University of California, Los Angeles, California 90095, United States; § Division of Chemistry and Chemical Engineering, 6469 California Institute of Technology, Pasadena, California 91125, United States; ∥ Molecular Foundry, 1666 Lawrence Berkeley National Laboratory, Berkeley, California 94720, United States

## Abstract

Topologically complex carbon nanostructures are an exciting but largely unexplored class of materials due to their challenging synthesis. Previous methods are low yielding because they rely on irreversible C_sp^2^_–C_sp^2^_ bond formation, which necessitates complex templating strategies to enforce entanglement. Here, reversible zirconocene coupling of alkynes is developed as a new method to access complex molecular topologies, where dynamic C–C bond formation facilitates entanglement under thermodynamic control, allowing the use of simple precursors without the need for preassembly. This strategy enables the scalable, high-yield synthesis of three topologically distinct nanocarbons, including the serendipitous discovery of a structure containing a new topological motif that was not previously identified or realized synthetically. This motif, consisting of an unusual combination of interlocking and interweaving, was recognized to be generalizable to a new topological class of molecules, introduced here as perplexanes.

## Graphical abstract

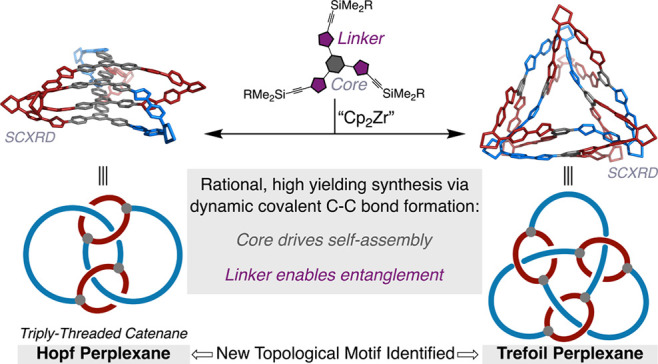

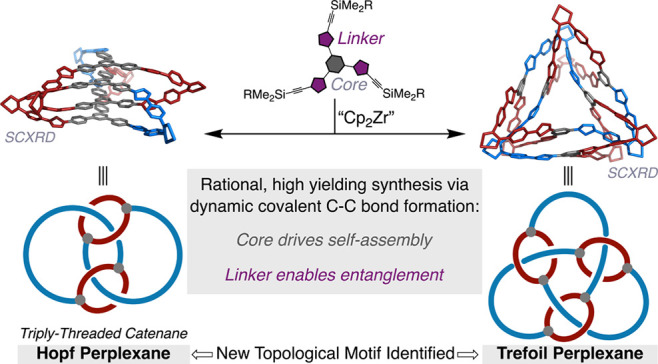

## Introduction

Topological complexity is ubiquitous in everyday life, where interlocking, braiding, and interweaving motifs underpin valuable technologies like textiles and building materials that demand strength and flexibility. Increasingly, topology is also being recognized as a critical design parameter on the nanoscale, where modes of entanglement in polymers and other extended systems have drastic effects on physical properties.^1–5^ Topological control on the molecular scale is also known to impart unique behaviors, including anion-binding and catalysis,^6^ photophysics,^7,8^ supramolecular chemistry,^9^ and membrane transport.^10^ However, molecular control of topology is still in its infancy, with only a range of relatively simple structures being accessible.^11,12^ To achieve precise, bottom-up control of topology at the molecular and nanoscale, expansion to new topologies and chemical functionalities are required.

Systems of particular interest are entangled nanocarbons, which represent a pinnacle of synthetic challenge in molecular topology. The inherent rigidity and lack of traditional functional handles in carbon-rich conjugated systems requires fundamentally new strategies for achieving topological complexity that push this field forward. Additionally, molecular nanocarbons share many of the desirable physical and electronic properties of related, extended nanomaterials (e.g., graphene and carbon nanotubes), as well as certain limitations such as poor solubility and processability. Encoding entanglement into these structures is a unique way to introduce flexibility, resulting in new dynamic behaviors and higher solubility.^13^

As evidence of this inherent challenge, to date only a few simple topologies for entangled nanocarbons have been realized (Figure 1a). These include Bäuerle’s oligothiophene [2]­catenanes obtained via copper-phenanthroline assemblies (9 steps, 5% yield),^14,15^ Cong’s [2]­catenane nanohoops prepared using copper coordination (7 steps, 8% yield, 13 mg)^16^ or azobenzene covalent templates (8 steps, 1.5% yield, 14 mg),^17^ Itami’s all-phenylene [2]­catenanes and trefoil knot via a cleavable spirosilane (7–8 steps, 0.1–2% yield, 0.8–2 mg)^13,18^ and Jasti’s nanohoop rotaxanes and catenanes, recently obtained with use of active metal templating (8 steps, 1.5% yield).^19^

**Figure 1:**
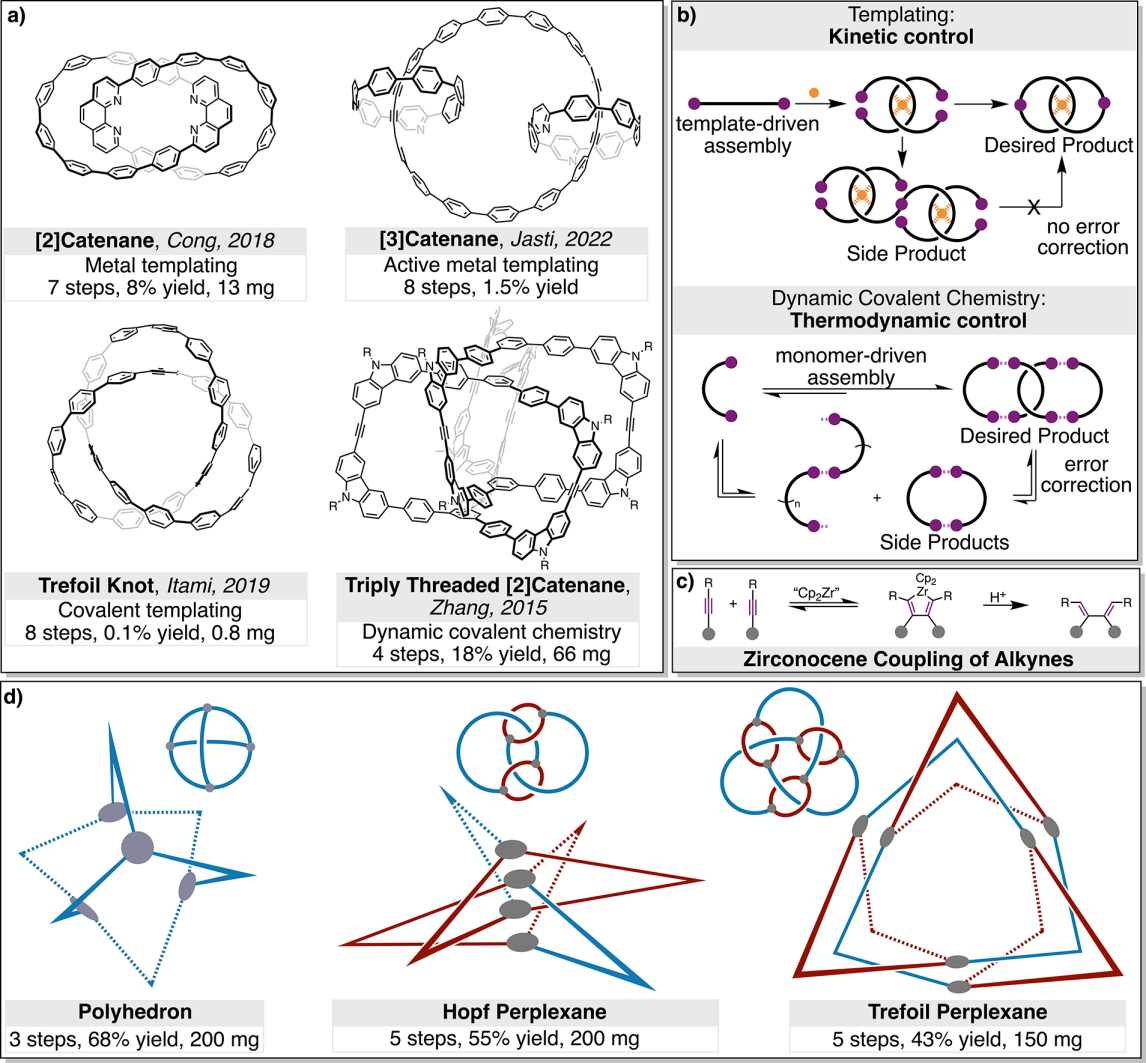
Summary of prior work on the synthesis of topologically complex nanocarbons and the advances introduced in this report. (a) Representative examples of reported topologies and synthetic strategies; (b) schematic highlighting the differences between kinetic and thermodynamic synthetic control; (c) the reversible C–C bond forming reaction type used in this work; and (d) the three topologies synthesized using this approach.

In each of these pioneering examples, C–C bond formation is achieved via irreversible coupling that does not provide a means for error correction (Figure 1b). This necessitates the use of strong directional templating to kinetically favor the entangled product, but such templates generally require several synthetic steps and highly specific functional groups. Together, this leads to multistep, low-yielding syntheses. In crafting an alternative to this approach, we were inspired by a prior report from Zhang and co-workers that utilizes alkyne metathesis on a tripodal building block to generate a “triply-threaded [2]­catenane” in high yield.^20^ Central to this approach is the use of dynamic C–C bond formation, allowing the reaction to occur under thermodynamic control. Due to this error correction mechanism, product formation can be efficiently driven by weaker forces like π-stacking, dispersion, and free-volume minimization that are more easily achieved with conjugated building blocks.

We also recognized that Zhang’s use of a branched monomer was key, enabling the formation of a densely entangled structure from a simple, symmetrical building block. Most entangled molecules are described as knots or links, mathematically well-defined topologies consisting of one or multiple interwoven and/or interlocked rings with a fixed number of crossing points. Increasing topological complexity has therefore focused largely on increasing the number of structural “crossings”, up to the current maximum of 12.^21–24^ Because this approach utilizes linear building blocks, increasing structural complexity usually requires increasingly complex monomers, straining the limits of synthetic chemistry. This makes the use of branched monomers highly desirable, because they are readily accessible and branching points rapidly expand potential topological complexity. The higher complexity associated with more branching components should allow access to new subclasses of topology that have not yet been mathematically identified, and the correspondingly vast structural space presents challenges to rational design. A few structural classes have been identified and synthesized, including Ravels^25,26^ and the “entangled polyhedra” developed by Fujita and co-workers,^27–29^ but examples are still limited and no nanocarbons of these types yet exist. Thus, the identification of new classes of branched topologies is poised to further expand the field, enabling the synthesis of more complex and chemically diverse structures from simpler building blocks.

Here we introduce a general strategy to access entangled nanocarbon cages using a reversible alkyne coupling to form C_sp^2^_–C_sp^2^_ bond linkages. This enables the high-yield formation of topologically complex structures in the absence of strong preorganization (Figure 1c). The size and topology of these structures can be rationally modified by monomer design, as illustrated below by the synthesis of three topologically distinct cage structures: a polyhedron, a triply interlocked catenane, and a wholly new topological construct comprising both interlocked and interwoven modes of entanglement (Figure 1d). The latter two structures are identified as members of a new, general topological class we dub perplexanes.

## Results and Discussion

Tripodal building blocks were chosen as the simplest branched units. Monomers were designed to assemble via only π-stacking and dispersion interactions, as these are the dominant forces in nanocarbon assembly. This assembly usually requires structural planarity in the monomer, which is somewhat at odds with the need for flexibility to accommodate entanglement. To balance these design elements, the targeted monomers have two conceptually distinct segments, dubbed “cores” and “linkers”. In this design, the core consists of a rigid, planar conjugated system that controls size and directs π-stacking/dispersion, while the linkers provide the required flexibility for interweaving. Conformational flexibility is achieved with the incorporation of a thiophene unit, which introduces a wide bite angle and can adopt a range of dihedral angles with respect to the core. The three examples presented here demonstrate the effectiveness of this design strategy by illustrating (1) the importance of flexible linkers in facilitating entanglement, and (2) that the π-stacking ability of the core dictates the structure and topology of the resulting product.

Zirconocene coupling was chosen as the C–C bond-forming reaction for several reasons. It is one of very few dynamic covalent reactions capable of forming C_sp^2^_–C_sp^2^_ bonds and has been used to synthesize a wide range of conjugated macrocycles in exceptional yield on large scale.^30^ Additionally, it has several distinct advantages over alkyne metathesis (the only comparable method) for topological nanocarbon synthesis. First, the reaction initially forms isolable metalated intermediates that are crystalline. This facilitates characterization via X-ray diffraction that is crucial for verifying topology, yet difficult to achieve for wholly organic compounds. Second, zirconocene coupling generates a bent rather than linear linkage, enabling the use of simple, planar building blocks.

First, an alkynylated triphenyltriazine (TPT) monomer **1-mon**, containing no flexible linker (Scheme 1), was investigated. It was anticipated that this monomer would form a simple polyhedron with no entanglement. Upon treatment with Cp_2_Zr­(Me_3_SiC≡CSiMe_3_)­(pyr) (a synthon for zirconocene, Cp_2_Zr)^30,31^ in tetrahydrofuran (THF), a complex mixture of products formed at 23 °C and persisted upon heating to 80 °C. However, after heating to 100 °C the mixture converged to a single major species, **1-Zr**, in 81% yield on a 200 mg scale. Changing the reaction solvent to benzene caused the same product to precipitate from the reaction mixture as single crystals. X-ray crystallography of **1-Zr** determined the structure to be a tetrameric polyhedron (Figure 2a,d). The **1-Zr** cage was quantitatively demetalated by treatment with HCl in benzene to provide the fully organic structure **1**. The retention of the polyhedral structure was confirmed by ^1^H NMR spectroscopy and MALDI–MS analysis (Supporting Information page S6 and S7). This cage topology mirrors the [4 + 6] cages synthesized by Fujita^32^ and Cooper^33^ via dynamic chemistry. However, while those approaches furnish structures with weak C-heteroatom or metal coordination bonds, **1** is connected by strong C–C bonds that display robust stability.

**Scheme 1:**
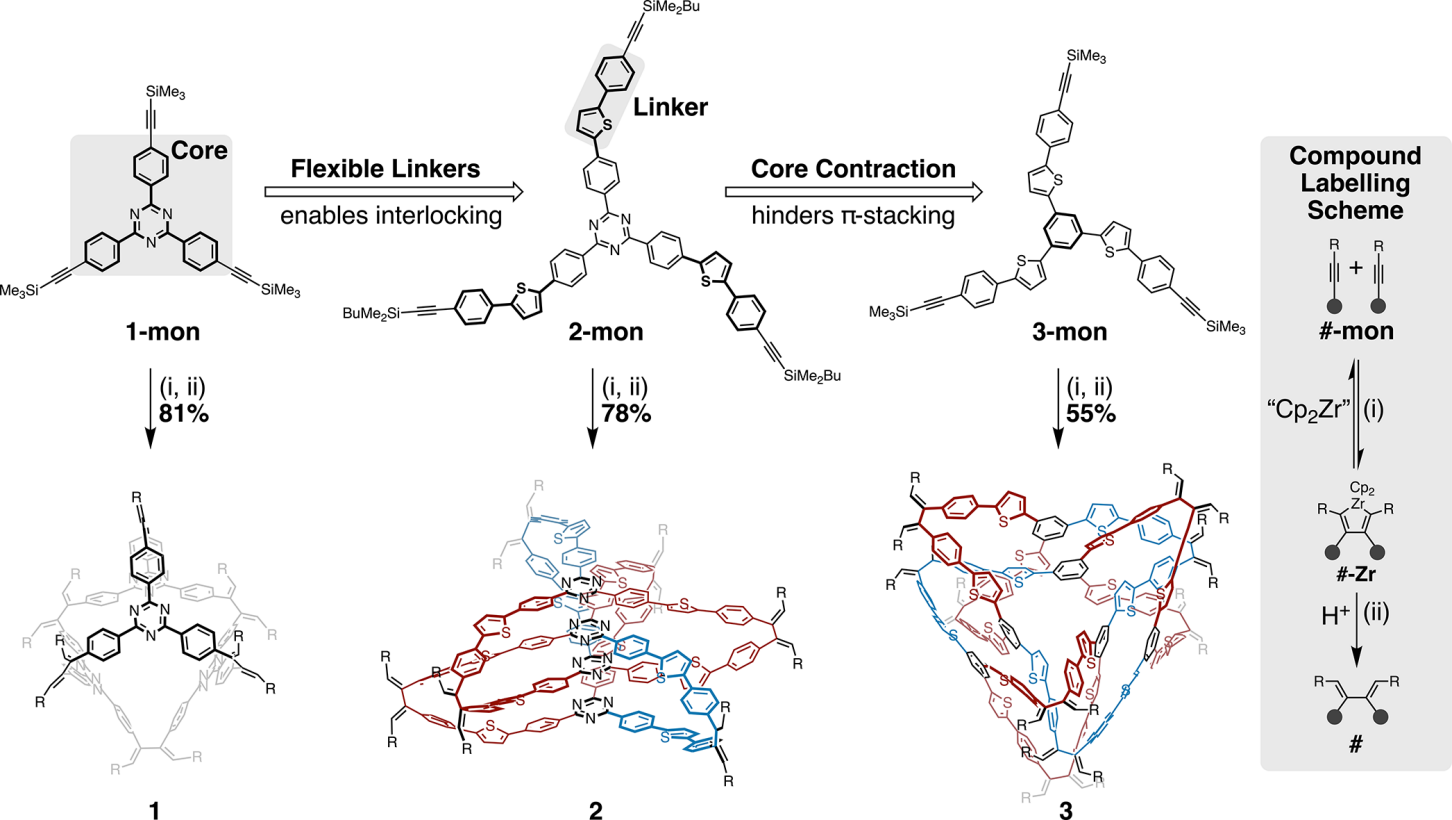
Synthesis of **1–3** ^a^

a Reagents and conditions (i) Cp_2_Zr­(Me_3_SiC≡CSiMe_3_)­(pyr), benzene, 60–100 °C. (ii) HCl, THF, 23 °C.

**Figure 2:**
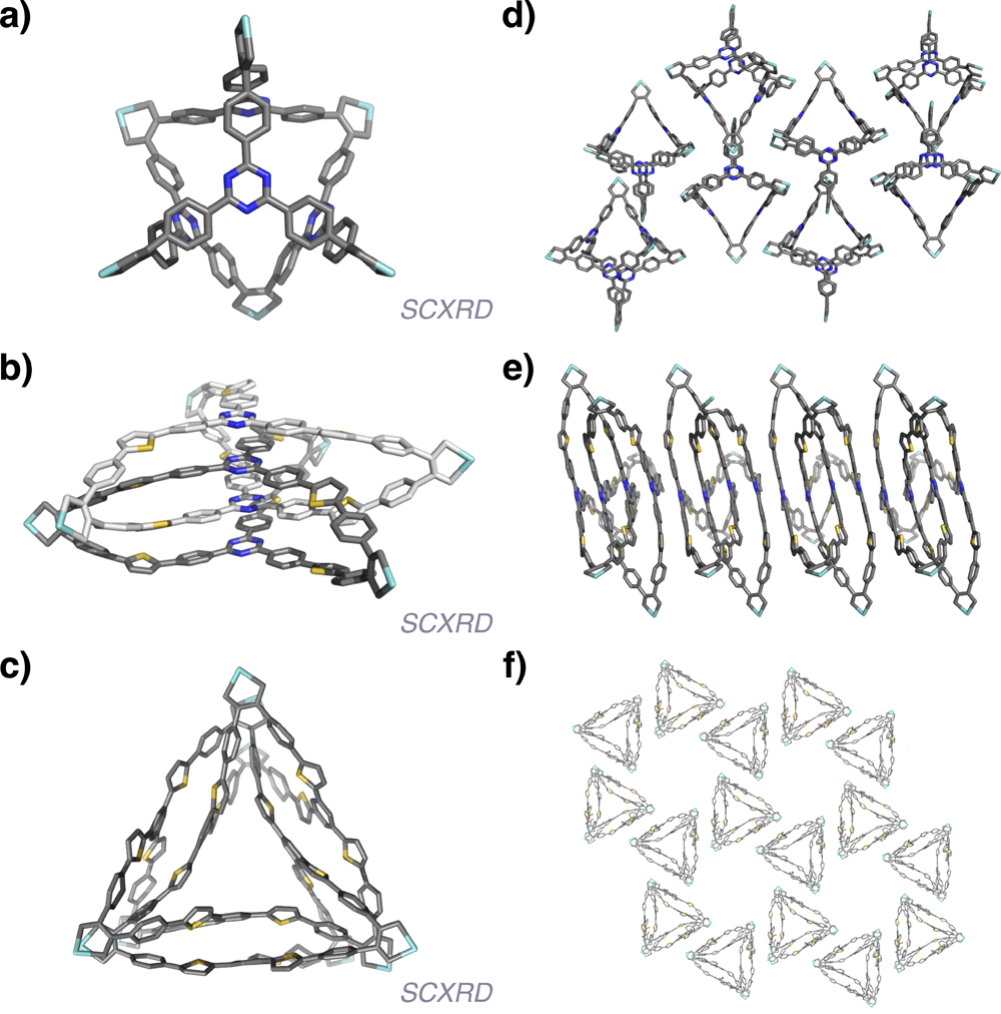
X-ray crystallographic structures of **1-Zr** (a,d), **2-Zr** (b,e), and **3-Zr** (c,f) with all side chains, solvent, and hydrogens truncated for clarity. (a–c) Individual cages; (d–f) solid-state packing.

Next, a triply threaded catenane was targeted as an archetypical example of an entangled cage to test the viability of the design strategy. The TPT core is well suited for this due to its propensity for π-stacking into well-defined aggregates.^34,35^ Monomer **2-mon** was generated by introducing the flexible thienylene-phenylene linker to the TPT core. Upon treatment with Cp_2_Zr­(Me_3_SiC≡CSiMe_3_)­(pyr) in THF, a single major species, **2-Zr**, formed rapidly upon heating to 60 °C, and was isolated in 78% yield on a 200 mg scale (Scheme 1). Product formation appears to be invariant to concentration, suggesting a significant enthalpic driving force. X-ray crystallography of **2-Zr** confirmed the triply interlocked catenane structure (Figure 2b,e). As with **1**, metal-free **2** was generated via quantitative demetalation as confirmed by MALDI–MS analysis and ^1^H NMR spectroscopy (Supporting Information page S7 and S8).

Analysis of the crystal structure of **2-Zr** highlights the role of both the core and linker in its assembly. Strong intramolecular π-stacking is observed between TPT cores, with an average distance of 3.3 Å, suggesting that catenane formation is largely driven by these core–core interactions. Compound **2-Zr** also forms ordered 1D columns in the solid state, driven by similarly strong intermolecular core–core π-stacking (Figure 2e). The linker accommodates this close packing of the cores by adopting a largely planar conformation, with an average thiophene-triazine dihedral angle of only ∼21°.

To quantify the strength of the monomer–monomer interactions in solution, the aggregation of a more soluble analog of **2-mon** with octyl instead of butyl chains, **2-mon-oct**, was studied by variable-concentration ^1^H NMR spectroscopy (see Supporting Information pages S14–S16 for details). The concentration of **2-mon-oct** was varied over an order of magnitude between 0.8 and 46 mM in benzene-*d*_6_ at 23 °C. The chemical shift of each aromatic peak was plotted as a function of concentration and fitted with good agreement to a monomer–dimer equilibrium model (Figure 3a). This model indicated *K*_a_ = 15 ± 2 M^–1^ and Δ*G* = −1.6 ± 0.7 kcal/mol (Figure 3b). Assuming no cooperative or anticooperative effects, this suggests that π-stacking provides an approximate 4.5 kcal/mol driving force for catenane assembly.

**Figure 3:**
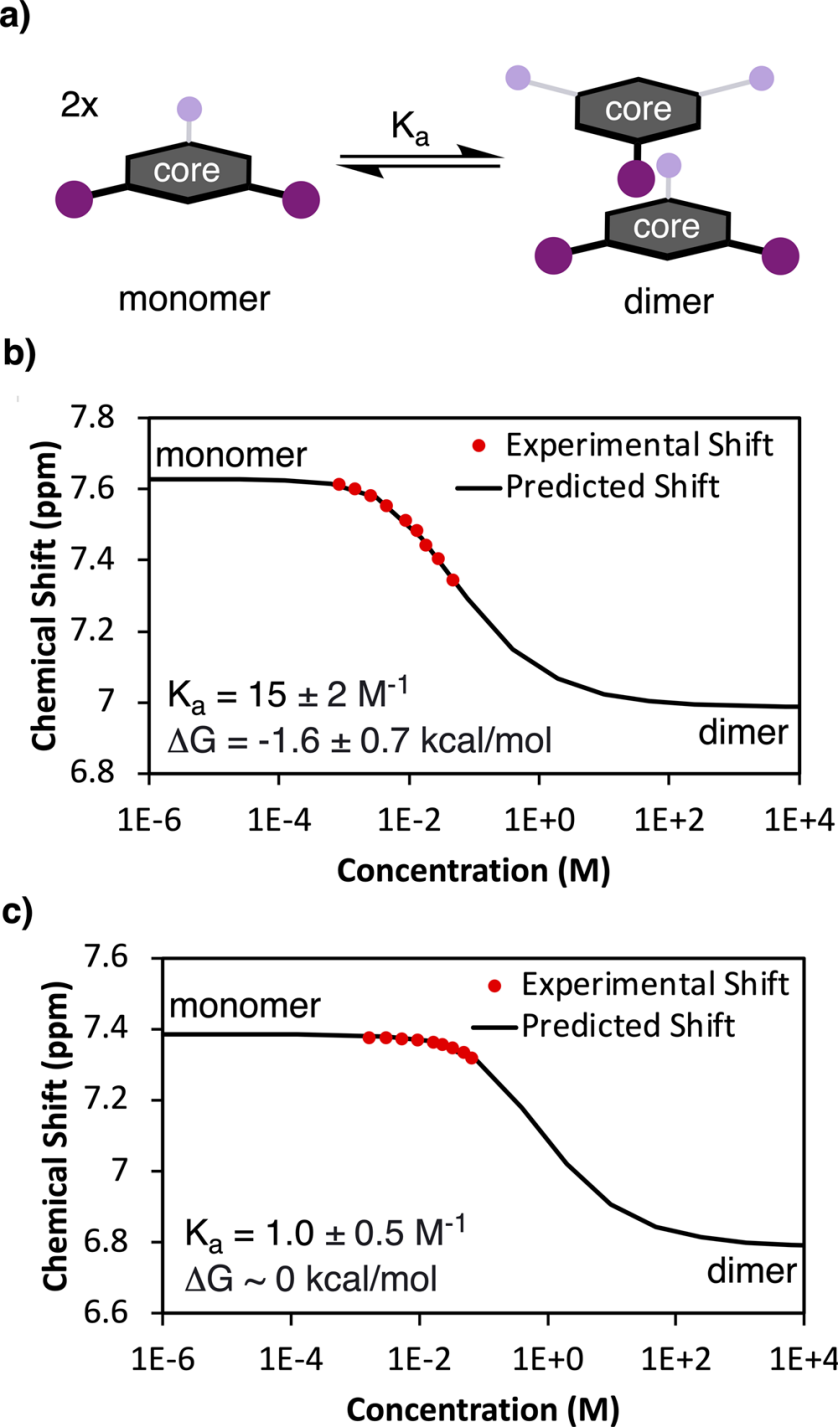
Fitting of variable concentration ^1^H NMR data to the monomer–dimer equilibrium model depicted in (a) for (b) **2-mon-oct**, and (c) **3-mon-oct**.

This efficient formation of π-stacked tetramers posed a design challenge for targeting other entangled structures. However, decreasing the size of the monomer core was expected to diminish π-stacking and geometrically inhibit the formation of an analogous tightly interlocked catenane, opening thermodynamic pathways to other structures. To achieve this, the TPT core was replaced by a trisubstituted benzene ring with the synthesis of **3-mon**.

Unlike that of **2-mon**, the reaction of **3-mon** with Cp_2_Zr­(Me_3_SiC≡CSiMe_3_)­(pyr) at 60 °C and 5.5 mM did not cleanly furnish a single product. The product formation is highly sensitive to concentration (see Supporting Information pages S11 for details), with higher concentrations favoring low-symmetry **3-Zr** (Scheme 1). At 56 mM, **3-Zr** was the sole well-defined product, and was isolated in 55% yield on a 150 mg scale. X-ray crystallographic analysis was accomplished in the same manner as for **2-Zr** and revealed the structure to be a highly entangled, fully continuous hexamer (Figure 2c,f). Compound **3** was generated from **3-Zr** under conditions identical to those used for **1** and **2**. Notably, while **3-mon** is insoluble in hexanes and only sparingly soluble in chlorinated solvents like DCM and chloroform, **3** is exceptionally soluble in hexanes (35 mg/mL) despite having a near-identical chemical composition and much higher molecular weight. This counterintuitive solubility highlights the unique dynamics of such densely intertwined structures.

In good agreement with expectations, there is no intramolecular π-stacking between monomers in the crystal structure, with an average core–core distance of 4.1 Å. This suggests that assembly is instead driven by weak dispersion interactions and a minimization of free volume. Similarly, the assembly of hexagonal arrays in the solid state appears to be driven only by weak intermolecular dispersion interactions (Figure 2f).

Variable concentration ^1^H NMR measurements of a soluble analog of **3-mon**, **3-mon-oct**, dissolved in benzene-*d*_6_, between 1.7 and 67 mM, corroborate the absence of strong attractive monomer interactions, with *K*_a_ = 1.0 ± 0.5 M^–1^ and Δ*G* ∼ 0 kcal/mol (Figure 3c). The formation of this highly complex hexameric structure in the absence of any templating or strong thermodynamic driving force highlights the utility of a dynamic approach to topological nanocarbon synthesis.

Remarkably, this combination of only dispersion interactions and free-volume minimization furnish a structure that is quite topologically complex. It is most simply visualized as a classical trefoil knot where each crossing point is exchanged for a threaded macrocycle. The introduction of branch points means that this structure cannot be topologically classified as a knot, yet it contains two trefoil knots of opposite handedness (rendering it a meso compound). Similarly, it contains rotaxane-like threaded macrocycles, yet they are interwoven into a single, fully covalent structure that does not meet the formal definition for a link or rotaxane. The structure can neither be described as an entangled polyhedron, as the core connectivity instead maps onto a simple cycle with embedded, smaller cycles.

The core topological motif of **3** is conceptually generalizable to any mathematical link by replacing each crossing point of the parent link with a threaded macrocycle such that the over-strand becomes the macrocycle, and the under-strand is threaded through it (Figure 4). Despite the fact that the common “triply-threaded catenane” topology is actually the simplest example of this motif, it has not been previously identified as a general topological class. Due to the lack of a well-recognized term for these structures, we propose the name perplexane, derived from the Latin word “perplexus” which means “entangled”. For an in-depth discussion of the underlying topology, see pages S16−18 in the Supporting Information.

**Figure 4:**
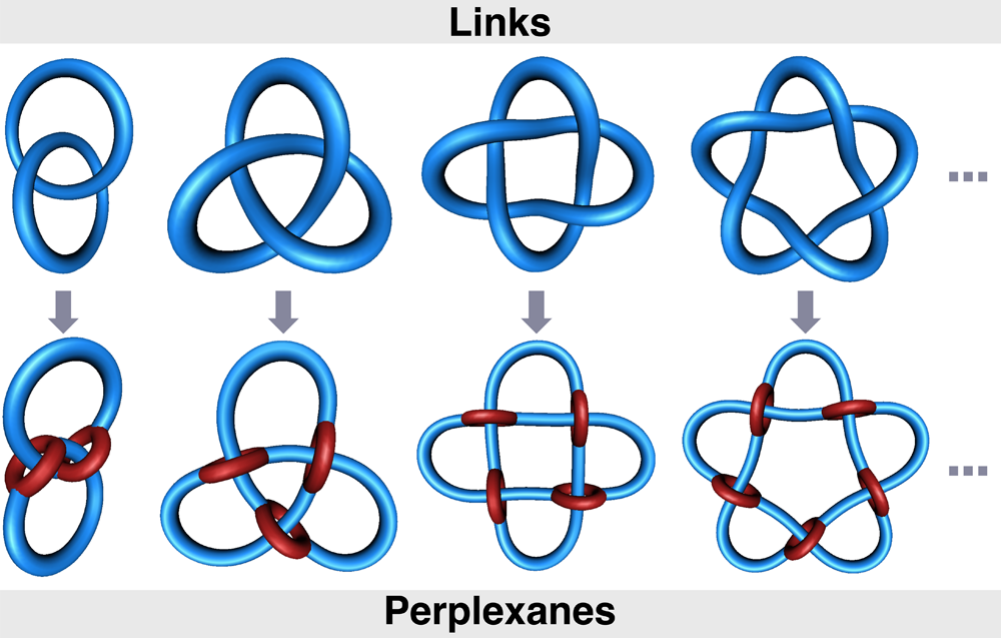
Conceptual derivation of perplexanes (bottom row) from four representative “parent” links via interconversion of crossing points to threaded loops (left to right): hopf link, trefoil knot, Solomon link, and pentafoil knot.

## Conclusion

This report introduces a new topological class of molecules, dubbed perplexanes, and details the synthesis of the two most fundamental examples of this subtype. The trefoil perplexane is the largest and most topologically complex nanocarbon synthesized to date and is accessed in unprecedented yield and scale due to the use of zirconocene coupling as a dynamic covalent C–C bond forming reaction. Perplexanes are expected to display dynamic and mechanical properties distinct from previously synthesized topologies due to their unique and dense pattern of entanglement, combining both interlocking and interweaving. The synthesis of higher order perplexanes remains a significant challenge that is likely to inspire further synthetic creativity and advances. We anticipate that the zirconocene-based methodology used here will play a key role in the synthesis of new nanocarbon topologies due to its ability to generate carbon rich structures in high yields under thermodynamic control and facilitate their crystallographic characterization. This combination of new conceptual targets and enabling methodology should engender new frontiers in topological nanocarbon synthesis.

## Supplementary Material



## Notes

General materials and methods, synthetic procedures, reaction optimization data, 1D and 2D nuclear magnetic resonance (NMR) characterization, matrix assisted laser desorption ionization (MALDI) mass spectrometry data, absorption and emission spectra, X-ray crystallography data and analysis, and detailed topological discussion (PDF)
