# Supplementary material for: Discovery of an Interlocked and Interwoven Molecular Topology in Nanocarbons via Dynamic C–C Bond Formation
Source: J Am Chem Soc. 2025 May 23;147(22):19132–8. doi: 10.1021/jacs.5c04268 (PMC12147124; doi:10.1021/jacs.5c04268)
Supplement: Supplementary file 1 [file ja5c04268_si_001.pdf]

Supporting Information for:

Discovery of an Interlocked and Interwoven Molecular Topology in Nanocarbons via Dynamic C-C Bond Formation

Harrison M. Bergman,[1] Angela T. Fan,[1] Christopher G. Jones,[2,3] August J. Rothenberger,[1] Kunal K. Jha,[3] Rex C. Handford,[1] Hosea M. Nelson,[2,3*] Yi Liu,[4*] T. Don Tilley[1*]

[1]Department of Chemistry, University of California, Berkeley, Berkeley, California 94720, United States

[2]Department of Chemistry and Biochemistry, University of California, Los Angeles, Los Angeles, California 90095, United States

[3]Division of Chemistry and Chemical Engineering, California Institute of Technology, Pasadena, California 91125, United States

[4]Molecular Foundry, Lawrence Berkeley National Laboratory, Berkeley, California 94720, United States

General details S1
Synthetic procedures and basic characterization data S2
Evidence that Product Formation of **3-Zr** is not Driven by Precipitation S10
Optimization of **3-Zr** Synthesis S11
Evidence for the Formation of 3-Zr Under Thermodynamic Control S12
^{1}H NMR Study of Monomer-Dimer Equilibria S14
In-Depth Topological Discussion of Perplexanes S16
^{1}H and ^{13}C{^{1}H} NMR Spectra S18
MALDI-TOF Spectrometry S31
Absorption and Emission spectroscopy S33
X-ray Crystallography S34
References S37

General Details

Unless otherwise stated, all manipulations were conducted in dry solvents under an inert atmosphere of nitrogen, using either standard Schlenk techniques or a glovebox. Pentane, toluene, tetrahydrofuran (THF), and diethyl ether, were dried using a JC Meyers Phoenix solvent purification system. Thiophene, hexamethyldisiloxane (HMDSO), C_6D_6, and $CDCl_3$ were freed from oxygen with vigorous nitrogen bubbling for 30 minutes, and then dried for at least 48 h over 3 Å molecular sieves (5% by mass). All reaction solvents were stored over 3 Å molecular sieves. **S1**,[1] **S3**,[2] **1-mon**,[3] and $Cp_2Zr(pyr)(Me_3SiC{\equiv}CSiMe_3)$[4] were synthesized by literature procedures. All other reagents were purchased from commercial suppliers and used as received. "Room temperature" or "RT" refers to ~22 ˚C. Reaction temperatures represent the oil bath temperature unless otherwise stated. Mass spectrometry of all compounds except **2** and **3** (see MALDI-TOF Spectrometry for details) was performed by the QB3/Chemistry Mass Spectrometry Facility at the University of California, Berkeley. Column chromatography was carried out using Fisher Chemical 40–63μm, 230–400 mesh silica gel. NMR (^{1}H and ^{13}C) spectra were obtained at room temperature on Bruker Avance 400, 500, and 600 MHz spectrometers. Chemical shifts (δ) are given in ppm and are referenced to residual solvent peaks for ^{1}H-NMR spectra (δ = 7.26 ppm for

$CDCl_3$, δ = 7.16 for C_6D_6, and δ = 3.62 for THF-*d8*) and ^{13}C-NMR spectra (δ = 77.16 ppm for $CDCl_3$, δ = 128.06 for C_6D_6, and δ = 67.21 for THF-*d8*).

Synthetic procedures and basic characterization data

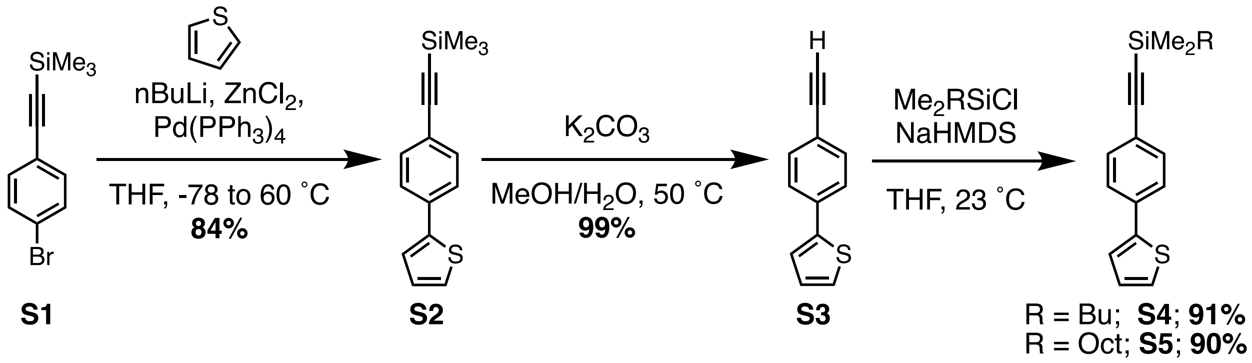

Scheme S1. Synthesis of linkers **S2**, **S4**, and **S5**.

S2. Degassed thiophene (4.65 g/4.43 mL, 0.055 mol, 1.4 eq) and THF (250 mL) was added to a dry schlenk flask under N_2. The reaction mixture was cooled to -78°C with a CO_2/isopropanol bath and n-BuLi (1.6 M in hexanes, 32.1 mL, 0.05 mol, 1.3 eq) was added via syringe to the solution over 10 minutes. After stirring for 10 minutes, $ZnCl_2$ (1.04 M in THF, 59.2 mL, 0.062 mol, 1.56 eq) was added via syringe over 2 minutes. The reaction mixture was then warmed back to room temperature. Once at RT, **S1** (10 g, 0.04 mol, 1 eq) and $Pd(PPh_3)_4$ (2.31 g, 0.002 mol, 0.05 eq) were added to the flask against the flow of N_2, heated at 65°C, and stirred for 12 h. The reaction mixture was then cooled to RT and quenched with aqueous ammonium chloride. This crude mixture was then extracted with CH_2Cl_2, dried over $MgSO_4$, and solvents were removed with rotary evaporation. The crude product was purified via filtration and a silica plug (20% DCM in hexanes) to afford **S2** as a colorless powder (7.7g, 84% yield). The ^{1}H NMR spectrum of this material matched the reported data.[2]

^{1}H NMR (400 MHz, $CDCl_3$) δ 7.55 (d, *J* = 8.5 Hz, 2H), 7.46 (d, *J* = 8.5 Hz, 2H), 7.33 (dd, *J* = 3.6, 1.1 Hz, 1H), 7.30 (dd, *J* = 5.1, 1.0 Hz, 1H), 7.08 (dd, *J* = 5.1, 3.6 Hz, 1H), 0.26 (s, 9H).

S4. To a 50 mL Schlenk flask was added **S3** (1.40 g, 7.60 mmol, 1.00 equiv) and THF (30 mL). A solution of sodium hexamethylsilazine (1.46 g, 7.98 mmol, 1.05 equivs) in THF (10 mL) was added slowly at RT and stirred for 10 min. Dimethylbutylchlorosilane (1.72 g, 11.4 mmol, 1.5 equivs) was subsequently added against N_2, and the reaction mixture was stirred for 1 h at RT. Aqueous ammonium chloride was then added and the reaction mixture was extracted with DCM (3x10 mL), dried with $MgSO_4$, and solvents were removed via rotary evaporation. The crude product was purified by column chromatography (100% hexanes) to afford **S4** (2.06 g, 91%) as a pale yellow oil. **^{1}H NMR (600 MHz, $CDCl_3$)** δ 7.58 – 7.52 (m, 2H), 7.49 – 7.44 (m, 2H), 7.33 (dd, *J* = 3.6, 1.2 Hz, 1H), 7.30 (dd, *J* = 5.1, 1.2 Hz, 1H), 7.09 (dd, *J* = 5.1, 3.6 Hz, 1H), 1.48 – 1.35 (m, 4H), 0.93 (m, 3H), 0.74 – 0.63 (m, 2H), 0.23 (s, 6H). **^{13}C NMR (151 MHz, $CDCl_3$)** δ 143.80, 134.49, 132.64, 128.31, 125.65, 125.55, 123.74, 122.27, 105.39, 94.77, 77.37, 77.16, 76.95, 26.40, 26.20, 16.06, 13.96, -1.53. **ESI-MS (m/z):** [M]+ calcd. for $C_{18}H_{22}SSi$, 298.1211; found, 298.1217.

S5. This compound was synthesized and purified according to the procedure outlined above for **S4** using the following amounts: **S3** (700 mg, 3.80 mmol) in 15 mL THF, sodium hexamethylsilazine (730 mg, 3.99 mmol) in 5 ml THF, Dimethyloctylchlorosilane (1.18 g, 5.70 mmol) to afford **S5** (1.21 g, 90%) as a viscous yellow oil. **^{1}H NMR (400 MHz, $CDCl_3$)** δ 7.57 – 7.51 (m, 2H), 7.48 – 7.43 (m, 2H), 7.33 (dd, *J* = 3.7, 1.2 Hz, 1H), 7.30 (dd, *J* = 5.1, 1.1 Hz, 1H), 7.08 (dd, *J* = 5.1, 3.6 Hz, 1H), 1.50 – 1.16 (m, 12H), 0.93 – 0.83 (m, 3H), 0.73 – 0.64 (m, 2H), 0.22 (s, 6H). **^{13}C NMR (151 MHz, $CDCl_3$)** δ 143.79, 134.48, 132.63, 128.29, 125.63, 125.53, 123.72, 122.27, 105.42, 94.79, 77.37, 77.16, 76.95, 33.43, 32.10, 29.48, 29.43, 23.99, 23.95, 22.85, 16.33, 14.27, -1.51. **ESI-MS (m/z):** [M]+ calcd. for $C_{22}H_{30}SSi$, 354.6270; found, 354.6269.

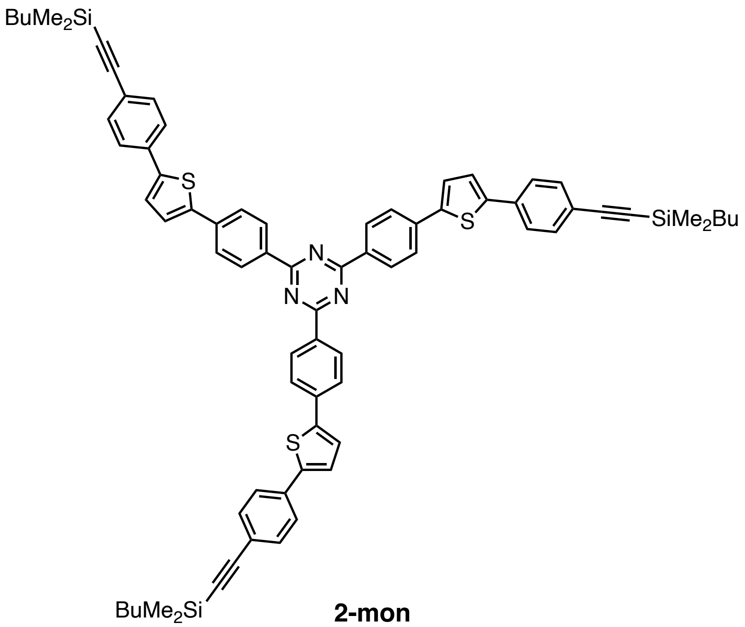

2-mon

2-mon. A 125 mL Schlenk flask was charged with **S4** (2.00 g, 6.70 mmol, 3.30 equiv) and tetrahydrofuran (25 mL) and the solution was cooled to –78 °C with a CO_2(s)/acetone bath. To this solution was added *n*-BuLi (1.85 M in hexanes, 3.99 mL, 6.50 mmol, 3.20 equiv) by syringe over 5 min and the resulting mixture was stirred for a further 1 h at –78 °C. To this mixture was added $ZnCl_2$ (1.05 M in THF, 6.57 mL, 6.90 mmol, 3.4 equiv) by syringe over 5 min, and subsequently the flask was removed from the cold bath and warmed to RT over 1 h. At this time *tris*(4-bromophenyl)-1,3,5-triazine (1.11 g, 2.03 mmol, 1.00 equiv) and $Pd(PPh_3)_4$ (234 mg, 0.203 mmol, 0.100 equiv) were added against a flow of N_2, the Schlenk flask was sealed, and the mixture was heated to 65 ˚C and stirred for 18 h. The solution was then exposed to air and diluted with aqueous ammonium chloride (40 mL). The crude product was extracted with CH_2Cl_2 (3x 20 mL), dried over $MgSO_4$, and solvents were removed by rotary evaporation. The crude product was purified by gradient column chromatography (from 15 to 40% CH_2Cl_2 in hexanes) and solvent was removed by rotary evaporation to afford **2-mon** (1.58 g, 65%) as a bright yellow powder. **^{1}H NMR (400 MHz, Chloroform-*d*)** δ 8.79 (d, *J* = 8.4 Hz, 6H), 7.83 (d, *J* = 8.4 Hz, 6H), 7.61 (d, *J* = 8.4 Hz, 6H), 7.50 (d, *J* = 8.3 Hz, 6H), 7.48 (d, *J* = 3.8 Hz, 3H), 7.37 (d, *J* = 3.8 Hz, 3H), 1.43 (dd, *J* =

7.8, 3.7 Hz, 12H), 0.93 (t, *J* = 6.9 Hz, 9H), 0.75 – 0.67 (m, 6H), 0.24 (s, 18H). **^{13}C NMR (151 MHz, CDCl$_3$)** δ 170.21, 143.55, 143.38, 137.41, 134.96, 134.09, 132.61, 129.34, 125.22, 125.14, 124.98, 124.74, 122.33, 105.49, 94.99, 77.37, 77.16, 76.95, 26.45, 26.25, 16.11, 14.02, -1.44. **MS-MALDI (m/z):** [M]+ calcd. for $C_{75}H_{75}N_3S_3Si_3$, 1197.4431; found, 1197.4427.

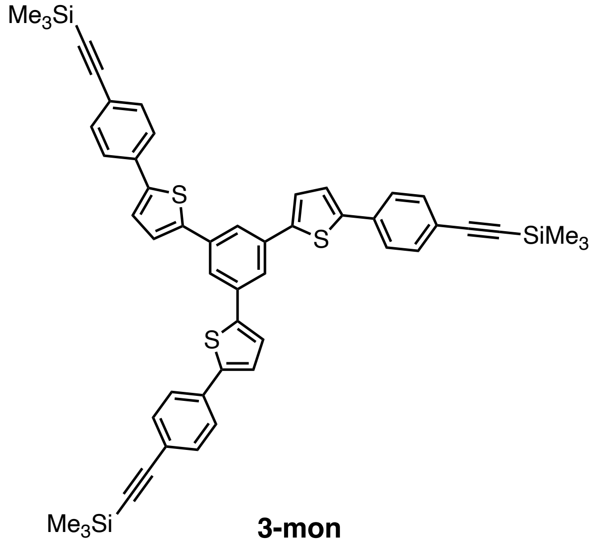

3-mon

3-mon. This compound was synthesized according to the procedure outlined above for **2-mon** using the following amounts: **S2** (4.00 g, 15.6 mmol, 3.50 equivs), nBuLi (1.85 M in hexanes, 8.27 mL, 15.3 mmol, 3.40 equivs), $ZnCl_2$ (1.05 M in THF, 15.6 mL, 16.2 mmol, 3.60 equivs), 1,3,5-tribromobenzene (1.42 g, 4.46 mmol, 1.00 equiv), $Pd(PPh_3)_4$ (578 mg, 0.50 mmol, 0.1 equiv) and 100 mL THF. The crude product was purified via column chromatography (20% DCM in hexanes), and subsequent precipitation from a saturated DCM solution with MeOH afforded **3-mon** (3.04 g, 81%) as a very faintly yellow-green powder. **^{1}H NMR (400 MHz, CDCl$_3$)** δ 7.77 (s, 3H), 7.64 – 7.56 (m, 6H), 7.53 – 7.46 (m, 6H), 7.42 (d, *J* = 3.8 Hz, 3H), 7.37 (d, *J* = 3.8 Hz, 3H), 0.27 (s, 27H). **^{13}C NMR (151 MHz, CDCl$_3$)** δ 143.65, 143.24, 135.69, 134.20, 132.71, 125.41, 125.09, 124.76, 122.42, 122.16, 105.00, 95.63, 77.37, 77.16, 76.95, 0.13. **MS-MALDI (m/z):** [M]+ calcd. for $C_{51}H_{48}S_3Si_3$, 840.2226; found, 840.2229.

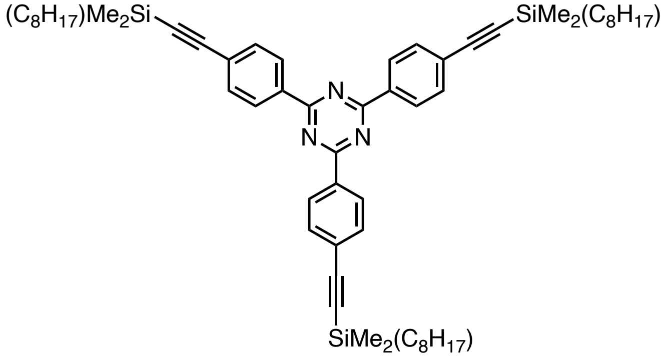

1-mon-oct

1-mon-oct. A flask was charged with *tris*(4-bromophenyl)-1,3,5-triazine (0.100 g, 0.183 mmol), $(PPh_3)_2PdCl_2$ (0.013g, 0.018 mmol), and copper(I) iodide (0.007 g, 0.037 mmol). Tetrahydrofuran

(5 mL) and triethylamine (2 mL) were then added, followed by ethynyl(dimethyl)octylsilane (0.126 g, 0.641 mmol). The reaction mixture was stirred at 60 °C for 18 h, then quenched with sat. NH_4Cl (5 mL) and extracted with dichloromethane (3 x 10 mL). The organic fractions were combined and dried with $MgSO_4$, then the solvent was evaporated and the residual solid dissolved in hexanes and eluted through a short plug of silica (10 g) with hexanes. The solvent was evaporated and the yellow solid was recrystallized from methanol to afford **1-mon-oct** as pale yellow crystals (0.134 g, 82%). **^{1}H NMR (600 MHz, $CDCl_3$)** δ = 8.68 (d, *J* = 8.4 Hz, 6H), 7.64 (d, *J* = 8.4 Hz, 6H), 1.46 (m, 6H), 1.39 (m, 6H), 1.31 (m, 24H), 0.89 (t, *J* = 6.9 Hz, 9H), 0.72 (m, 6H), 0.27 (s, 18H) . **^{13}C NMR (151 MHz, $CDCl_3$)** δ = 171.22, 135.90, 132.38, 128.88, 127.67, 105.19, 97.24, 33.43, 32.11, 29.49, 29.44, 23.99, 22.85, 16.26, 14.28, -1.57. **MS-MALDI (m/z):** [M]+ calcd. for $C_{57}H_{81}N_3Si_3$, 891.5738; found 891.61.

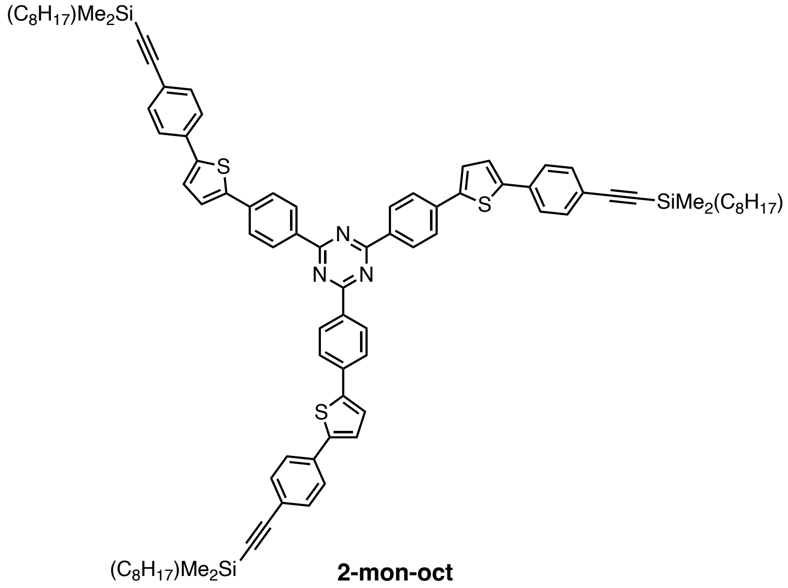

2-mon-oct

2-mon-oct. This compound was synthesized according to the procedure outlined above for **2-mon** using the following amounts: **S5** (500 mg, 1.36 mmol, 3.20 equiv), nBuLi (1.85 M in hexanes, 0.708 mL, 1.31 mmol, 3.10 equivs), $ZnCl_2$ (1.05 M in THF, 1.33 mL, 1.40 mmol, 3.3 equivs), *tris*(4-bromophenyl)-1,3,5-triazine (231 mg, 0.424 mmol, 1.00 equiv), $Pd(PPh_3)_4$ (49.0 mg, 0.042 mmol, 0.10 equiv), and 6 mL THF. The crude product was purified via column chromatography (25% DCM in hexanes) afford **2-mon-oct** (423 mg, 73%) as a bright yellow powder. **^{1}H NMR (500 MHz, $CDCl_3$)** δ 8.74 (d, *J* = 8.3 Hz, 6H), 7.82 – 7.75 (m, 6H), 7.61 – 7.56 (m, 6H), 7.49 (d, *J* = 8.2 Hz, 6H), 7.44 (d, *J* = 3.8 Hz, 3H), 7.34 (d, *J* = 3.7 Hz, 3H), 1.51 – 1.18 (m, 36H), 0.89 (t, *J* = 6.7 Hz, 9H), 0.75 – 0.67 (m, 6H), 0.25 (s, 18H). **^{13}C NMR (151 MHz, $CDCl_3$)** δ 170.38, 143.69, 143.40, 137.55, 135.05, 134.10, 132.64, 129.43, 125.26, 125.21, 125.11, 124.78, 122.40, 105.48, 95.06, 77.37, 77.16, 76.95, 33.49, 32.14, 29.53, 29.48, 24.03, 22.88, 16.39, 14.30, 2.81, -1.43. **MS-MALDI (m/z):** [M]+ calcd. for $C_{87}H_{99}N_3S_3Si_3$, 1365.6309; found 1365.6320.

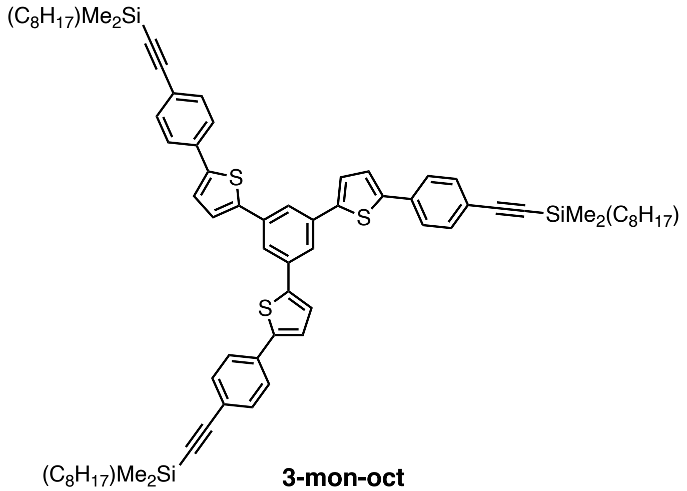

3-mon-oct

3-mon-oct. This compound was synthesized according to the procedure outlined above for **2-mon** using the following amounts: (500 mg, 1.36 mmol, 3.20 equiv), nBuLi (1.85 M in hexanes, 0.708 mL, 1.31 mmol, 3.10 equivs), $ZnCl_2$ (1.05 M in THF, 1.33 mL, 1.40 mmol, 3.3 equivs), 1,3,5-tribromobenzene (133 mg, 0.424 mmol, 1.00 equiv), $Pd(PPh_3)_4$ (49.0 mg, 0.042 mmol, 0.10 equiv), and 6 mL THF. The crude product was purified via column chromatography (15% DCM in hexanes) to afford **3-mon-oct** (366 mg, 76%) as a very faintly yellow-green powder. **^{1}H NMR (600 MHz, $CDCl_3$)** δ 7.57 (s, 3H), 7.55 – 7.51 (m, 6H), 7.48 (d, *J* = 8.3 Hz, 6H), 7.24 (d, *J* = 3.7 Hz, 3H), 7.22 (d, *J* = 3.8 Hz, 3H), 1.56 – 1.27 (m, 36H), 0.94 (t, *J* = 6.9 Hz, 9H), 0.81 – 0.71 (m, 6H), 0.30 (s, 18H). **^{13}C NMR (151 MHz, $CDCl_3$)** δ 143.29, 143.09, 135.30, 134.11, 132.64, 125.25, 124.80, 124.58, 122.38, 121.55, 105.45, 95.03, 77.37, 77.16, 76.95, 33.46, 32.12, 29.51, 29.46, 24.01, 22.86, 16.36, 14.29, 2.80, -1.47. **MS-MALDI (m/z):** [M]+ calcd. for $C_{72}H_{90}S_3Si_3$, 1134.5512; found, 1134.5518.

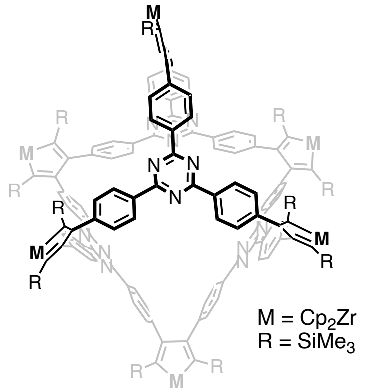

1-Zr

1-Zr. A Schlenk flask was charged with **1-mon**, (200 mg, 0.334 mmol) and Rosenthal's complex, $Cp_2Zr(pyr)(Me_3SiC{\equiv}CSiMe_3)$, (260 mg, 0.502 mmol). Benzene (3 mL) was then added and the dark purple solution was heated to 100 ^{o}C while stirring for 16 h. The heterogeneous reaction mixture was cooled to room temperature and the precipitate recovered by filtration. The resulting solid was washed with pentane (3 x 5 mL) to yield the product as a fine yellow powder (0.264 g, 85%). **^{1}H NMR (600 MHz, THF-d_8)** δ = 8.25 (d, *J* = 8.25 Hz, 24H), 6.90 (d, *J* = 8.25 Hz, 24H),

6.40 (s, 60H), -0.35 (s, 108H). **^{13}C NMR (151 MHz, THF-*d*$_8$)** δ = 206.64, 171.16, 151.45, 150.10, 133.55, 130.56, 128.13, 112.47, 67.39, 25.31, 2.88.

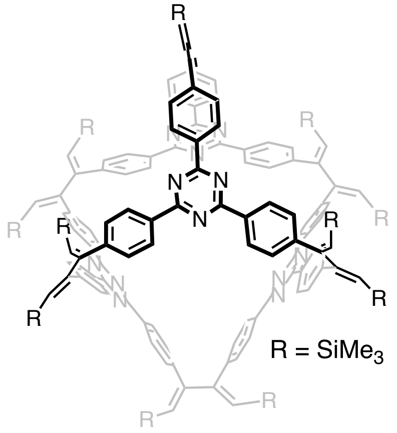

1

1. Tetrahedron **1-Zr** (0.240 g, 0.064 mmol) was dissolved in THF (5 mL) and to this solution was added 2 M HCl in dioxane (0.4 mL, 0.8 mmol). The reaction mixture was diluted with sat. Na_2CO_3 (1 mL) then the product extracted with dichloromethane (3 x 5 mL). The organic layers were combined, dried with $MgSO_4$, and the solvent evaporated. The residual solid was washed with MeOH (5 mL) and EtOH (5 mL) to recover the product as a white solid (0.150 g, 98%). **^{1}H NMR (600 MHz, $CDCl_3$)** δ = 8.41 (d, *J* = 8.4 Hz, 24H), 7.18 (d, *J* = 8.4 Hz, 24H), 6.40 (s, 12H), -0.04 (s, 108H). **^{13}C NMR (151 MHz, $CDCl_3$)** δ = 170.43, 160.67, 146.05, 134.50, 132.19, 129.70, 127.99, 0.47. **MS-MALDI (m/z)** [M]+ calcd. for $C_{144}H_{168}N_{12}Si_{12}$, 2401.0746; found 2401.11.

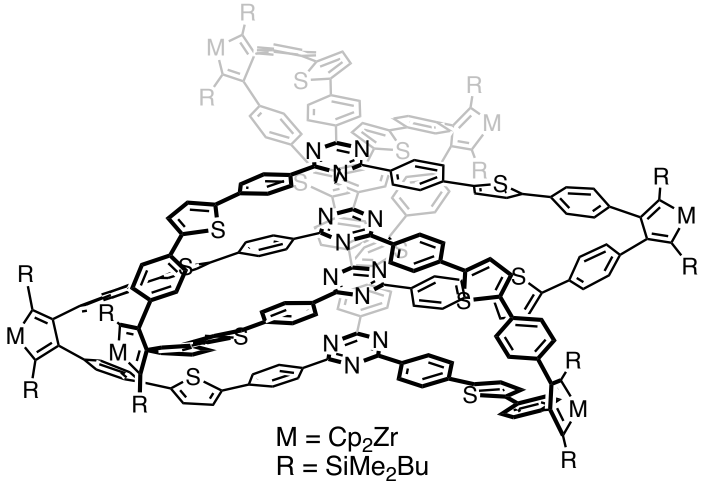

2-Zr

2-Zr. A Teflon stoppered flask was charged with **2-mon** (283 mg, 0.237 mmol, 1.00 equiv) and Rosenthal's complex, $Cp_2Zr(pyr)(Me_3SiC{\equiv}CSiMe_3)$, (169 mg, 0.358 mmol, 1.51 equiv). Benzene (7 mL) was then added, and the flask was sealed and heated to 60 ˚C for 16 h. The reaction mixture was cooled to RT and the crystalline precipitate was collected and washed with benzene (3x2 mL) to afford **2-Zr** (290 mg, 80%) as yellow crystals. **^{1}H NMR (400 MHz, $CDCl_3$)** The ^{1}H NMR of these single crystals (Figure S22) is complex. While the number of Cp and alkylsilane resonances

match the expected symmetry of the structure as determined by X-ray crystallography, the aromatic region contains only six sharp resonances with the expected integrations (half the expected 12) in addition to several broad and several small, sharp resonances. Despite this, the material was used directly in the subsequent step and cleanly furnishes demetalated **2**. This suggests that the unexpected complexity of the aromatic region is a result of slow dynamic conformational changes on the NMR timescale due to the relative inflexibility of **2-Zr** compared to **2**.

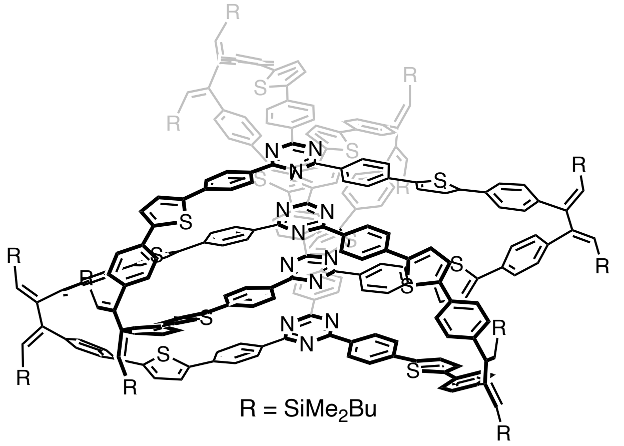

2

2. A 25 mL Schlenk flask was charged with **2-Zr** (273 mg, 0.045 mmol, 1.00 equiv), suspended in THF (5 mL), and stirred at RT. Trifluoroacetic acid (128 mg, 1.125 mmol, 25.0 equiv) was added dropwise via syringe over 1 min, during which time the solution became homogeneous. The reaction mixture was quenched with aqueous $NaHCO_3$ (5 mL), extracted with DCM (3x5 mL), and concentrated to dryness via rotary evaporation. The resulting crude solid was dissolved in DCM (20 mL), precipitated via addition of MeOH (40 mL), and collected by vacuum filtration. The solid was washed with MeOH (3x5 mL) and dried to afford **2** (204 mg, 96%) as a bright yellow powder. **^{1}H NMR (400 MHz, CD_2Cl_2)** δ 7.99 (d, *J* = 8.0 Hz, 12H), 7.48 (d, *J* = 7.9 Hz, 12H), 7.38 (d, *J* = 7.8 Hz, 12H), 7.30 (d, *J* = 7.9 Hz, 12H), 7.19 (d, *J* = 8.0 Hz, 12H), 7.09 (dd, *J* = 12.7, 8.0 Hz, 24H), 7.02 – 6.92 (m, 12H), 6.72 (d, *J* = 7.8 Hz, 12H), 6.52 (d, *J* = 1.7 Hz, 12H), 6.19 (d, *J* = 3.7 Hz, 6H), 5.97 (d, *J* = 3.7 Hz, 6H), 1.46 – 1.38 (m, 24H), 1.35 (dt, *J* = 7.6, 4.0 Hz, 24H), 1.02 – 0.95 (m, 18H), 0.91 (q, *J* = 6.3, 5.6 Hz, 18H), 0.67 (d, *J* = 8.2 Hz, 12H), 0.56 (d, *J* = 9.5 Hz, 12H), 0.10 (s, 36H) 0.00 (s, 36H). **^{13}C NMR (151 MHz, CD_2Cl_2)** δ 170.18, 169.38, 161.29, 144.87, 143.47, 142.92, 142.47, 142.23, 141.66, 137.73, 137.00, 134.90, 134.29, 133.12, 132.75, 131.31, 131.17, 129.36, 129.10, 128.97, 128.60, 125.47, 125.35, 125.12, 124.61, 124.48, 124.26, 124.06, 123.76, 72.90, 71.66, 62.21, 54.36, 54.18, 54.00, 53.82, 53.64, 43.82, 30.27, 27.27, 27.17, 26.90, 26.83, 17.11, 17.01, 14.29, 14.21, 1.34, -1.02, -1.15. **MS-MALDI (m/z):** [M]+ calcd. for $C_{300}H_{312}N_{12}S_{12}Si_{12}$, 4801.86; found 4801.86.

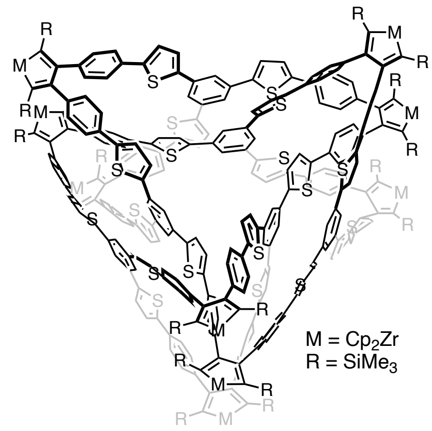

3-Zr

3-Zr. This compound was synthesized according to the procedure outlined above for **2-Zr** using the following amounts: **3-mon** (295 mg, 0.350 mmol, 1.00 equiv), Rosenthal's complex, $Cp_2Zr(pyr)(Me_3SiC{\equiv}CSiMe_3)$, (249 mg, 0.529 mmol, 1.51 equiv), benzene (6.5 mL), and a reaction time of 72 h to afford **3-Zr** (230 mg, 56%) as a yellow crystalline solid. **^{1}H NMR (600 MHz, THF-d_8)** δ 7.55 – 7.48 (m, 12H), 7.46 (dd, *J* = 8.1, 1.9 Hz, 6H), 7.41 (dd, *J* = 7.9, 2.0 Hz, 6H), 7.35 (dd, *J* = 8.0, 2.0 Hz, 6H), 7.26 (d, *J* = 3.6 Hz, 6H), 7.24 (d, *J* = 3.7 Hz, 6H), 7.15 (d, *J* = 1.6 Hz, 6H), 7.12 (d, *J* = 3.8 Hz, 6H), 7.11 – 7.06 (m, 18H), 7.04 (d, *J* = 8.0 Hz, 6H), 7.03 – 6.98 (m, 12H), 6.94 (dd, *J* = 8.1, 1.7 Hz, 6H), 6.88 – 6.83 (m, 12H), 6.82 (t, *J* = 1.6 Hz, 3H), 6.73 (d, *J* = 1.6 Hz, 6H), 6.67 (s, 30H), 6.65 (t, *J* = 1.6 Hz, 3H), 6.53 (s, 30H), 6.47 (s, 30H), 6.15 (d, *J* = 4.0 Hz, 3H), 5.80 (d, *J* = 4.0 Hz, 3H), 0.14 (s, 27H), -0.05 (s, 54H), -0.07 (s, 27H), -0.14 (s, 54H).

Note: Unlike for **2-Zr**, the concentration plays an important role in product distribution, with higher concentrations favoring the selective formation of **3-Zr**. The concentration used here (54 mM) gave the best results, as discussed in more detail on page S11.

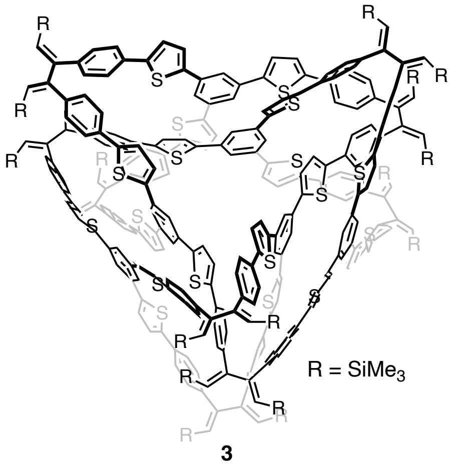

3

3. This compound was synthesized according to the procedure outlined above for **2** using the following amounts: **3-Zr** (215 mg, 0.031 mmol, 1.00 equiv), trifluoroacetic acid (88 mg, 0.78 mmol, 25 equiv), and THF (4 mL) to afford **3** (152 mg, 98%) as an off-white powder. **^{1}H NMR (600 MHz, THF-d_8)** δ 7.51 (d, *J* = 8.1 Hz, 6H), 7.46 (d, *J* = 8.1 Hz, 12H), 7.44 – 7.37 (m, 18H), 7.30 (d, *J* = 8.2 Hz, 18H), 7.25 – 7.21 (m, 12H), 7.21 – 7.16 (m, 12H), 7.14 – 7.05 (m, 18H), 6.98 – 6.97 (m, 3H), 6.95 – 6.94 (m, 6H), 6.78 – 6.77 (m, 3H), 6.75 – 6.73 (m, 12H), 6.68 – 6.63 (m,

6H), 6.49 (d, J = 10.5 Hz, 12H), 6.15 (d, J = 4.1 Hz, 3H), 5.83 (d, J = 4.1 Hz, 3H), 0.26 (s, 27H), 0.16 (s, 54H), 0.09 (s, 81H). **^{13}C NMR (151 MHz, THF)** δ 160.61, 160.32, 159.85, 159.55, 141.05, 140.92, 140.82, 140.65, 140.31, 140.24, 140.07, 140.02, 139.18, 139.04, 138.74, 138.60, 132.15, 132.09, 131.98, 131.76, 131.25, 131f.19, 131.13, 131.02, 129.30, 128.68, 128.47, 128.27, 128.06, 123.37, 122.33, 122.10, 121.88, 121.74, 121.59, 121.43, 121.35, 121.17, 121.12, 120.42, 119.70, 116.53, 116.03, 65.05, 64.90, 64.84, 64.69, 64.55, 64.40, 64.26, 27.79, 22.93, 22.80, 22.74, 22.67, 22.60, 22.47, 22.34, 22.20, 20.35, 11.51, -1.85, -2.00, -2.09, -2.18. **MS-MALDI (m/z):** [M]+ calcd. for $C_{306}H_{306}S_{18}Si_{18}$, 5059.47; found 5059.47.

Evidence that Product Formation of 3-Zr is not Driven by Precipitation

Many thermodynamically controlled syntheses of macrocycles and topologically complex molecules rely on precipitation of the product to increase yield via Le Chatelier's principle.[5-7] Although our synthesis uses product crystallization as a convenient means of *in-situ* isolation and purification of the desired products, it is not the thermodynamic driving force for the high yields observed. To confirm this, we generated **3-Zr** in THF rather than benzene, in which the product does not precipitate. *In-situ* ^{1}H NMR of this reaction mixture (Figure S1) illustrates that the product forms in comparable yield to the isolated yields reported in benzene, showing that the selectivity observed is inherent to the thermodynamic favorability of product formation in solution.

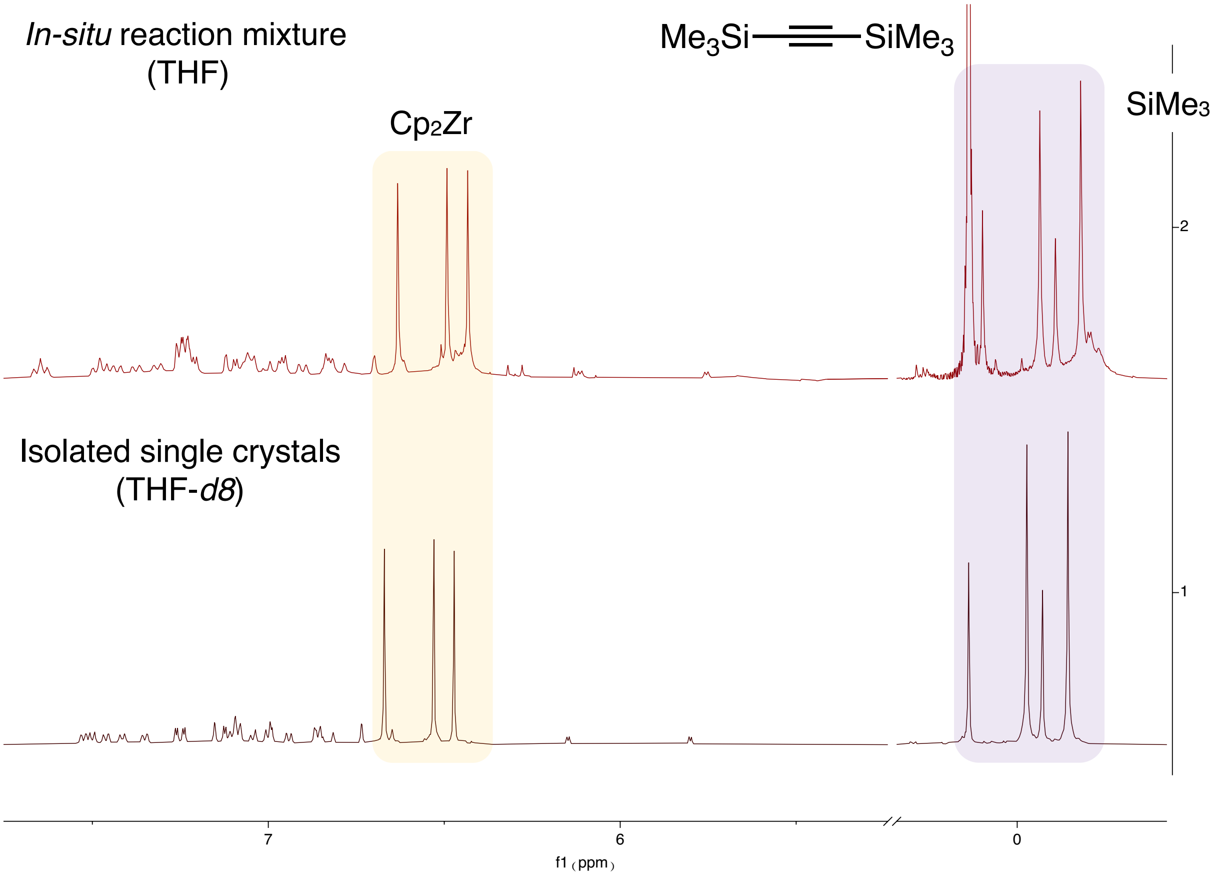

Figure S1. ^1H NMR spectra (500 MHz) of **3-Zr**, comparing an *in-situ* reaction mixture in THF (top) to isolated single crystals from a reaction in benzene redissolved in THF-*d8* (bottom).

Optimization of 3-Zr Synthesis

3-Zr was synthesized at a range of concentrations over one order of magnitude and monitored *in-situ* by ^1H NMR (Figure S2) to determine the effect of concentration on product distribution. Although benzene was used as the reaction solvent for isolation, we conducted these experiments in THF due to its ability to fully solubilize the reaction mixture even at elevated concentration. As evidenced in the previous section, product distribution and yield appear to be similar between benzene and THF. *In-situ* yield was determined by integration of the product Cp peaks against the bis(trimethylsilyl)acetylene released by Rosenthal's complex as an internal standard. This study clearly illustrates that **3-Zr** is formed more selectively and in higher yield as concentration is increased, ostensibly due to the decrease in entropic penalty for the assembly of six monomer units. This suggests that the competing side products are smaller than **3-Zr**.

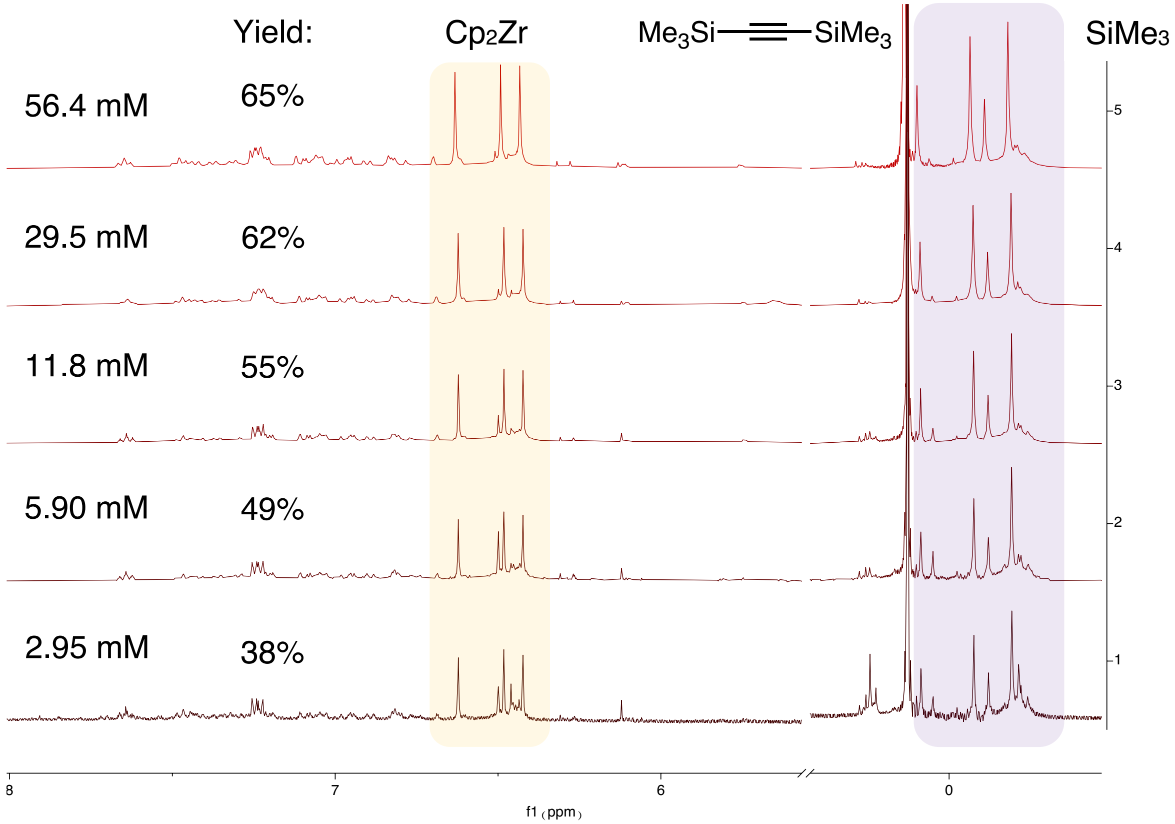

Figure S2. ^1H NMR spectra (500 MHz, THF-*d8*) of in-situ reaction mixtures of **3-Zr** at various concentrations from 2.95 – 56.4 mM to illustrate the effect of monomer concentration on yield and product distribution.

Evidence for the Formation of 3-Zr Under Thermodynamic Control

There is ample literature precedent for the dynamic nature of zirconocene coupling of alkynes,[8] as well as clear experimental indicators that this specific transformation is occurring under thermodynamic control. The region of the ^{1}H NMR spectrum between 6.2–6.8 ppm in THF is indicative of zirconacyclopentadiene species, which are the products of successful alkyne coupling. When monitoring the reaction of **3-mon** to **3-Zr** under optimized conditions (56.4 mM, THF, 60 ˚C), all Rosenthal's complex is consumed to form a mixture of zirconacyclopentadiene-containing species within the first 16 h. During the next 48 h, product concentration increases as side-product decreases (Figure S3). This is only possible via the breaking and reforming of zirconacycle linkages, indicating that the reaction is dynamic under optimized conditions. This trend is even more noticeable at lower concentrations (Figure S4). Furthermore, **3-Zr** generated at 60 ˚C can be subjected to elevated temperature (THF, 100 ˚C) for 2 h and a change in product distribution is observed (Figure S5). Notably, the most prominent side product is the same as the one observed when the reaction is run at low concentration (Figure S2). This is consistent with expectations for a reaction under thermodynamic control, where elevated temperature and lower monomer concentration both increase the entropic cost of assembling large oligomers and favor smaller products.

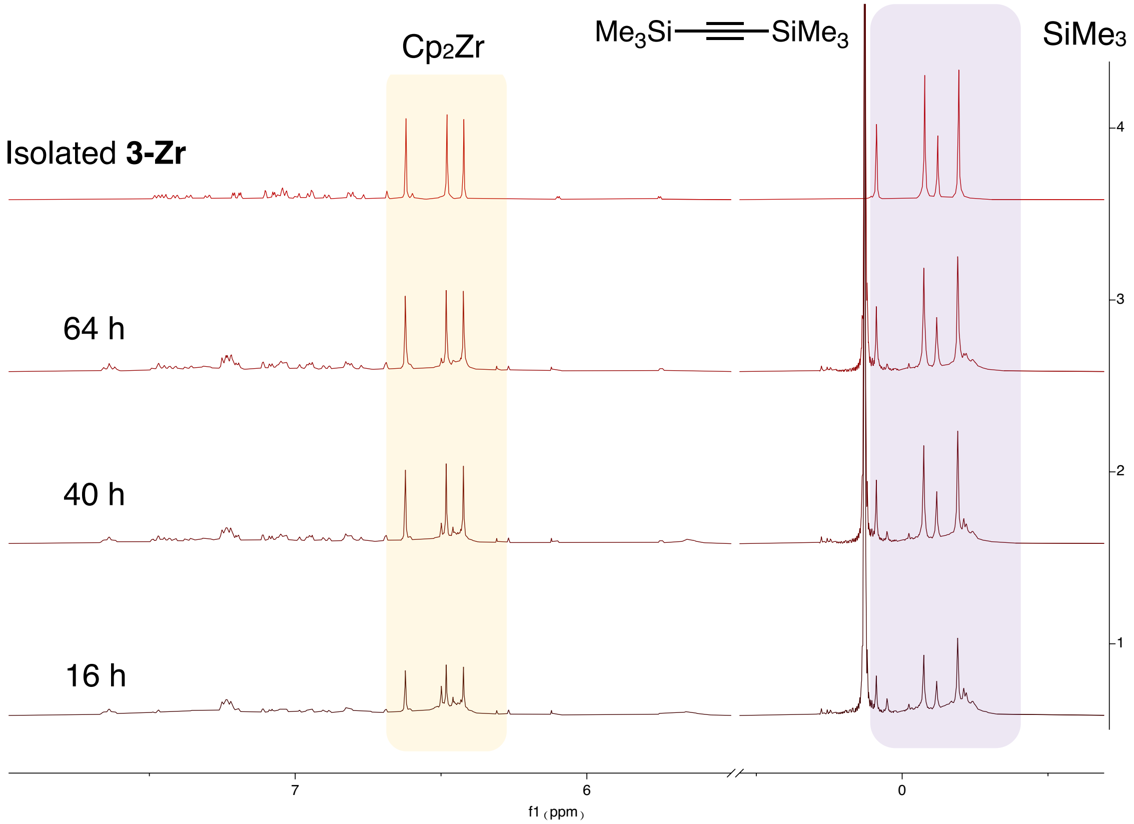

Figure S3. ^{1}H NMR spectra (500 MHz, THF-*d8* or THF) of in-situ reaction mixtures of **3-Zr** run at 56.4 mM and 60 ˚C at various time points to illustrate the change in product distribution over the course of the reaction.

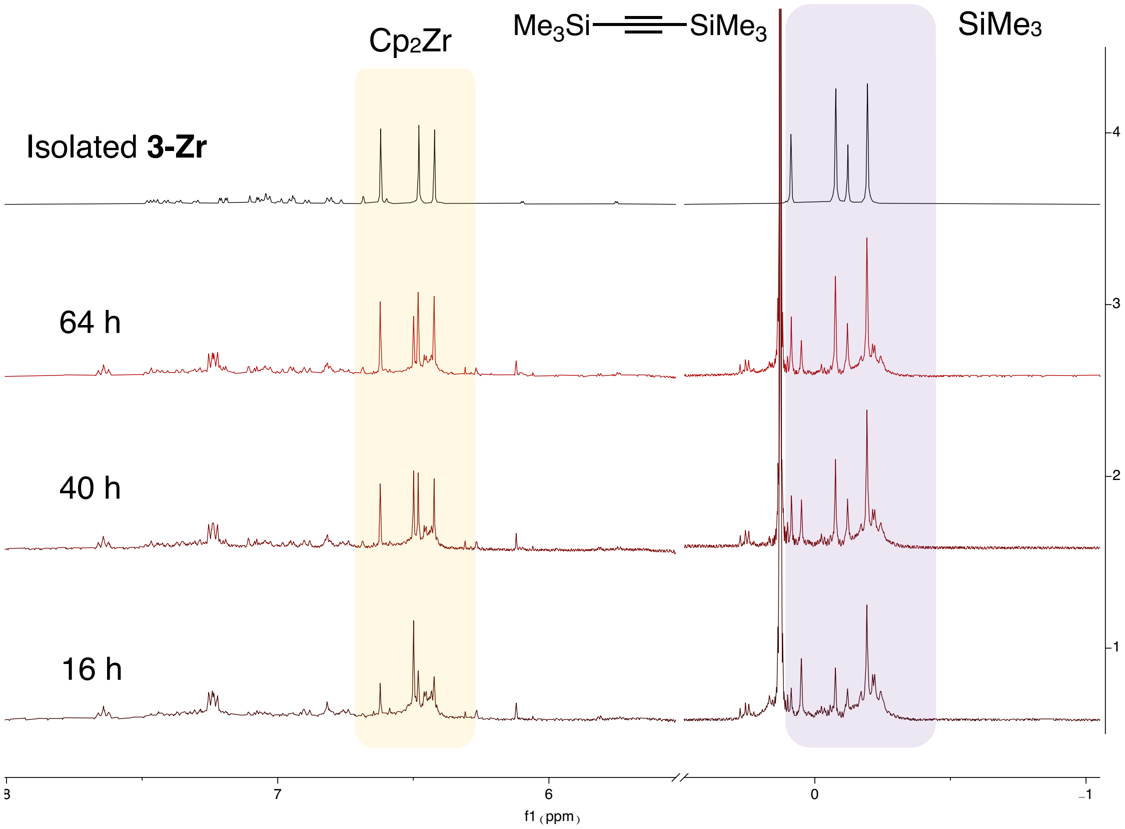

Figure S4. ^{1}H NMR spectra (500 MHz, THF-*d8* or THF) of in-situ reaction mixtures of **3-Zr** run at 5.90 mM and 60 ˚C at various time points to illustrate the change in product distribution over the course of the reaction.

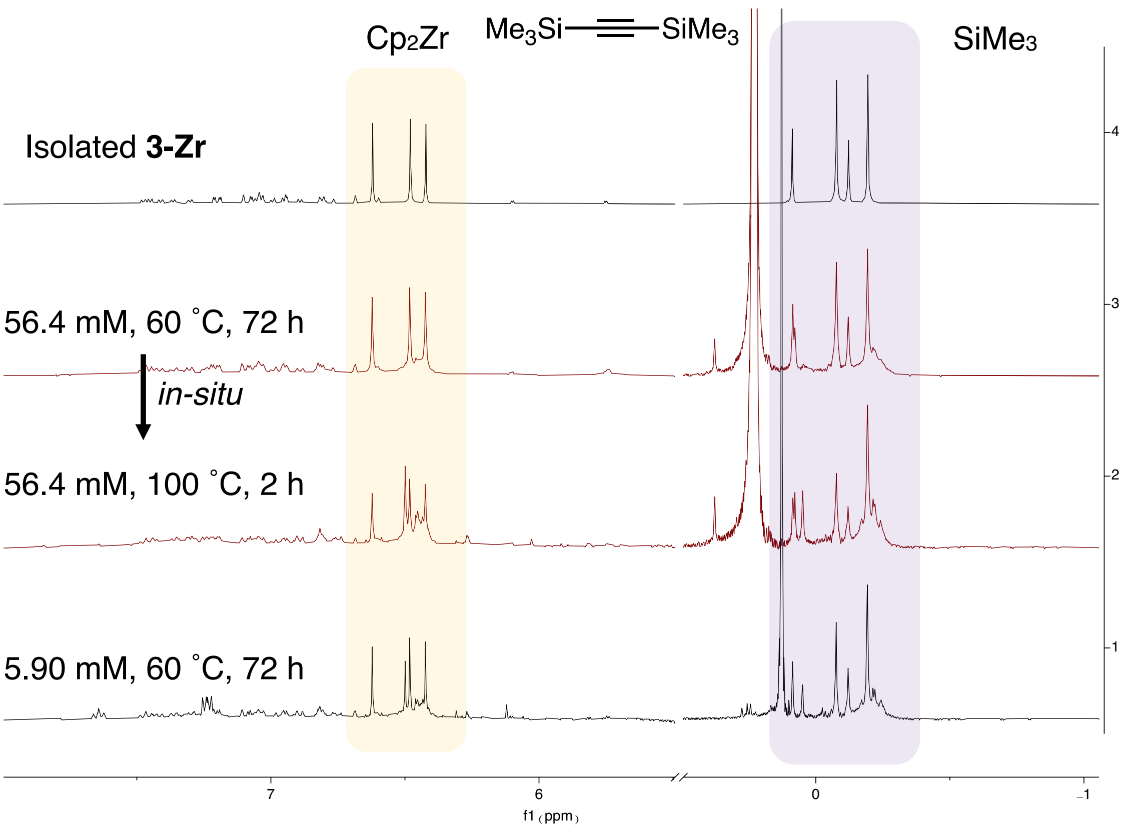

Figure S5. ^{1}H NMR spectra (500 MHz, THF-*d8* or THF) of in-situ mixtures of **3-Zr** run at 5.90 mM and 60 ˚C at various time points to illustrate the change in product distribution over the course of the reaction.

<u>^{1}H NMR Study of Monomer-Dimer Equilibrium in 1-mon-oct, 2-mon-oct, and 3-mon-oct</u>

Monomer-dimer equilibria of **1-mon-oct**, **2-mon-oct**, and **3-mon-oct** were investigated by variable concentration ^{1}H NMR spectroscopy in benzene to mimic the conditions used for product isolation of **1-Zr**, **2-Zr**, and **3-Zr** respectively. Maximum concentrations were set by solubility limits of each monomer. Chemical shifts of all aromatic resonances were averaged, this average was plotted as a function of concentration, and the data was fitted to a monomer-dimer equilibrium model via the regression approach developed by Moore and coworkers.[9-10] The supporting information of reference 10, pages S9-18 provides a detailed description of the monomer dimer model used here, as well as our general approach to regression analysis. Briefly, initial values for chemical shift of the monomer (δ_{mon}), chemical shift of the dimer (δ_{dim}), and equilibrium constant (K_{eq}) were chosen and fed into the previously developed monomer-dimer model. These values were used to generate predicted chemical shifts (δ_p) at each experimental concentration and compared to the experimental shifts (δ_{exp}) to calculate a standard deviation (σ). New values for the three variables were guessed recursively to achieve a local minimum using a Generalized Reduced Gradient Nonlinear solver in Microsoft Excel, and the resulting K_{eq} was taken and used to calculate

ΔG°. The raw data, final variable values, and predicted chemical shifts are summarized in the tables S1-S6.

**Concentration** (M)	δ_{exp} (ppm)	δ_p (ppm)
0.0782	8.143	8.142
0.0580	8.148	8.148
0.0387	8.153	8.153
0.0258	8.156	8.156
0.0172	8.159	8.159
0.0098	8.161	8.161
0.0049	8.162	8.162
0.0025	8.163	8.163
0.0012	8.163	8.163

Table S1. Experimental concentrations and chemical shifts (δ_{exp}) of **1-mon-oct** from variable concentration ^{1}H NMR (500 MHz, C_6D_6) compared to predicted chemical shifts (δ_p) from the monomer–dimer equilibrium model.

δ_{mon} (ppm)	δ_{dim} (ppm)	K_{eq}	ΔG° (kcal/mol)	σ
8.163	7.041	0.1240	-1.24	0.0037

Table S2. Optimal monomer–dimer model values to minimize standard deviation between experimental and predicted values for **1-mon-oct**.

**Concentration** (M)	δ_{exp} (ppm)	δ_p (ppm)
0.0464	7.346	7.348
0.0281	7.408	7.403
0.0182	7.443	7.448
0.0129	7.487	7.482
0.0088	7.515	7.515
0.0046	7.556	7.559
0.0025	7.582	7.586
0.0015	7.603	7.603
0.0008	7.617	7.613

Table S3. Experimental concentrations and chemical shifts (δ_{exp}) of **2-mon-oct** from variable concentration ^{1}H NMR (500 MHz, C_6D_6) compared to predicted chemical shifts (δ_p) from the monomer–dimer equilibrium model.

δ_{mon} (ppm)	δ_{dim} (ppm)	K_{eq}	ΔG° (kcal/mol)	σ
7.628	6.987	14.82	-1.60	0.0039

Table S4. Optimal monomer–dimer model values to minimize standard deviation between experimental and predicted values for **2-mon-oct**.

**Concentration** (M)	δ_{exp} (ppm)	δ_p (ppm)
0.0673	7.320	7.320
0.0495	7.336	7.334
0.0330	7.349	7.349
0.0233	7.358	7.358
0.0168	7.362	7.365
0.0095	7.370	7.373
0.0053	7.373	7.377
0.0031	7.374	7.380
0.0017	7.376	7.381

Table S5. Experimental concentrations and chemical shifts (δ_{exp}) of **3-mon-oct** from variable concentration ^{1}H NMR (500 MHz, C_6D_6) compared to predicted chemical shifts (δ_p) from the monomer–dimer equilibrium model.

δ_{mon} (ppm)	δ_{dim} (ppm)	K_{eq}	$\Delta G°$ (kcal/mol)	σ
7.384	6.788	1.002	-0.0012	0.0037

Table S6. Optimal monomer–dimer model values to minimize standard deviation between experimental and predicted values for **3-mon-oct**.

In-Depth Topological Discussion of Perplexanes

In claiming to require a new topological descriptor for the synthesized perplexanes, it is important to highlight why existing descriptors do not adequately describe these structures.

First, this issue is complicated by the existence of two different but often conflated topological formalisms: mathematical topology and chemical topology. This friction arises for two reasons. The first is because there are many objects whose topologies are mathematically trivial (i.e. not meaningfully distinct), yet as molecules they display distinct properties and structures that are important to describe and catalog. An illustrative example is the rotaxane, a structure in which a circle is threaded by a linear axle, which cannot be dethreaded due to the presence of bulky stoppers. In mathematics, this structure is topologically meaningless; the size of the circle is not defined by the topology, so if the circle is large enough it can always dethread. Yet in chemistry this motif effectively interlocks two molecular components, and it is a valuable endeavor to understand how different imposed topologies affect the properties of rotaxanes. The second is because mathematical topology is well developed to discuss certain types of topological complexity (particularly knotting and braiding) but does not contain good descriptive nomenclature for a large swathe of potential entangled structures. Thus, there are some instances

for which the types of molecules accessible to chemists are not easily described by existing mathematical topology. In these cases, chemists often develop colloquial names that are more facile for describing new structures within the community. Catenanes are good examples of this, in that they are mathematically links, but the wide range of linking patterns for even a handful simple rings are not simply delineated by existing nomenclature. Chemists often find it easier to catalog molecular links using the [n]catenane nomenclature, where "n" denotes the number of interlocked components, and simple additional descriptions, such as "radial", "linear", or "branched" catalog different linking patterns.[11] When we discuss a new topological class, it is important to distinguish between its mathematical descriptors, which provide a crucial theoretical framework for thinking about topology, and its chemical descriptors, which catalog important structural features in a way that facilitates better chemical understanding.

For the proposed perplexanes, we make the case that they do not fit into existing mathematical descriptions of knots or links yet contain mathematically non-trivial features, and further, that although the family is connected by an obvious and intuitive structural motif, different members of the family contain distinct underlying topological features. Due to the absence of an existing mathematically rigorous name, and to the structural similarity of this family despite subtly different underlying topologies, we feel that the development of a new chemical topology descriptor is the most useful way to classify these molecules. Below, evidence for these claims is presented.

First, the existence of branching points (in this case tripodal vertices) formally precludes these structures from being knots or links, even if they may contain these elements. This is because the formal definition of a knot is "the embedding of a circle in three-dimensional Euclidean space", meaning they must consist of a single closed loop. Branched, entangled structures are broadly defined in mathematics as tangled graphs, as identified and developed by Hyde and coworkers.[12,13] This topological class comprises all graphs (2- or 3-dimensional collections of vertices connected by edges) that contain topologically non-trivial features, such as knots, links, or other entanglements. Due to the vast structural space this encompasses, only a very small number of descriptive subclasses have been identified, none of which apply to perplexanes. One subclass in this family, Ravels, were formally developed in 2008,[14] and experimentally realized in molecules in 2019.[7] Ravels, however, are not global topological features as is the case for knots and links, but are localized around vertices. In ravels, each arm of a given vertex entangles the other arms, as illustrated effectively in reference 14. The topological features of perplexanes are not localized around their vertices, confirming that they are not Ravels.

The mathematically non-trivial features of perplexanes consist of the larger features of the parent knot or link, in addition to the rotaxane-like interlocking of the additional introduced cycles. Unlike in rotaxanes, however, this interlocking is topologically non-trivial because it can only be disentangled by breaking the cycle. Because of this, link-derived perplexanes (e.g. Hopf and

Solomon) contain two distinct modes of interlocking, while knot-derived perplexanes (e.g. trefoil and pentafoil) contain both interlocking and interweaving motifs. Notably, while standard knots and links become disentangled with a single strand cleavage, perplexanes require at least two.

From this line of reasoning, it is clear that perplexanes contain a unique set of topological features not previously described mathematically or observed in chemistry. Their simple derivation from classical topological knots and links makes them objects of interest for further chemical investigation, warranting their identification as a new topological class.

^{1}H, ^{13}C{^{1}H}, and Selected 2D NMR spectra

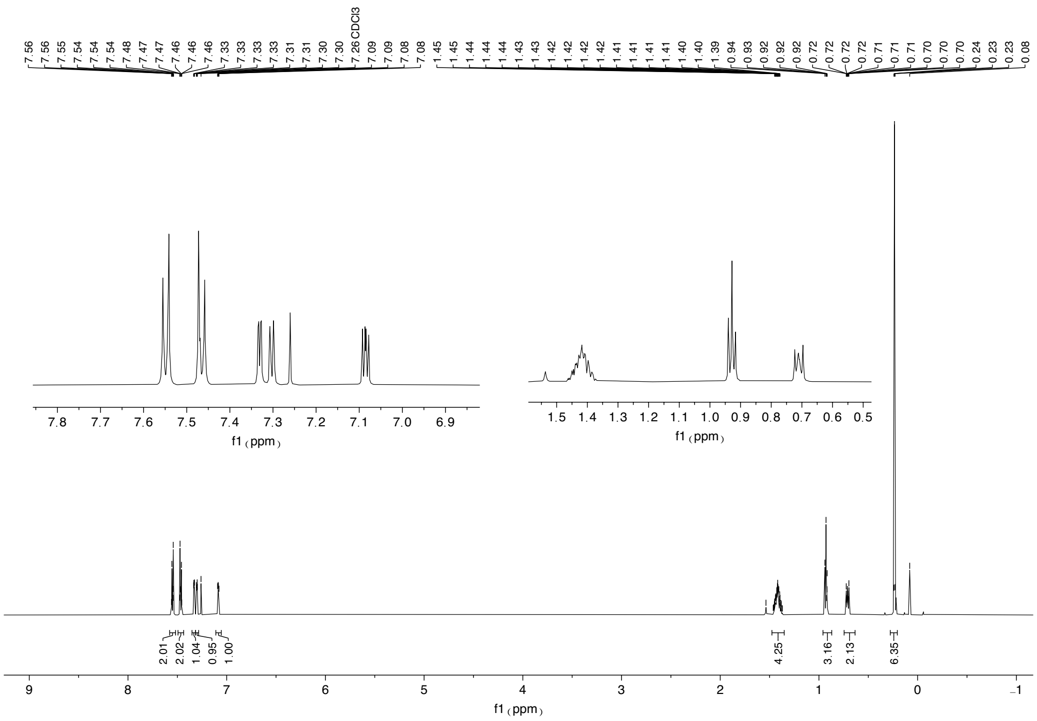

Figure S6. ^{1}H NMR Spectrum (400 MHz, $CDCl_3$) of **S4**.

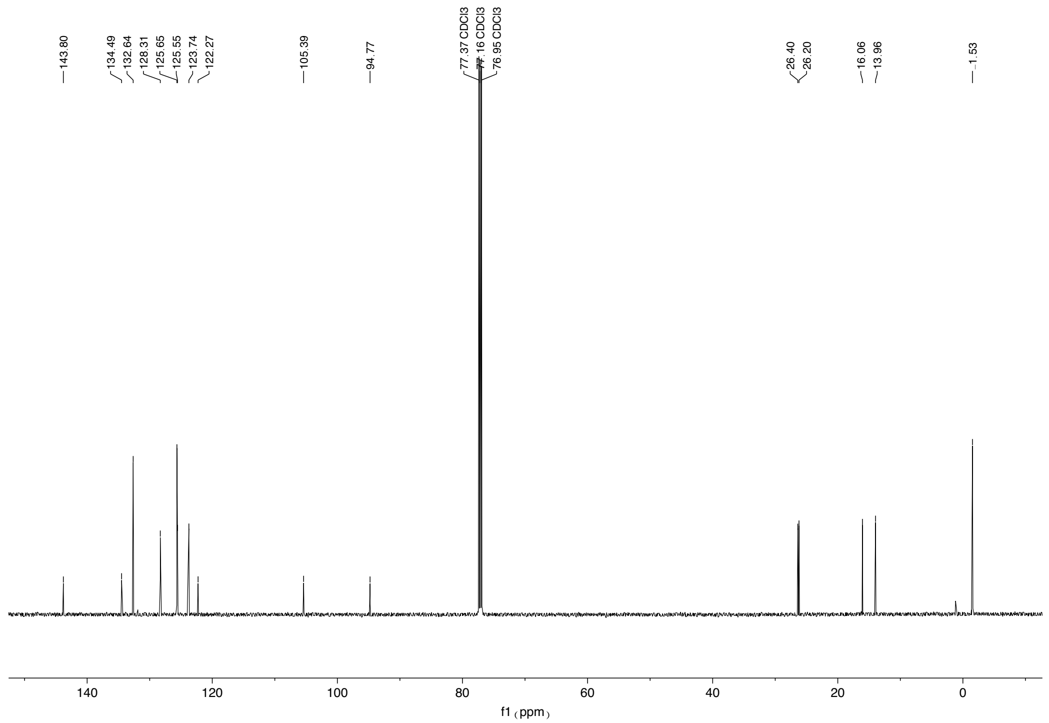

Figure S7. $^{13}C\{^{1}H\}$ NMR Spectrum (151 MHz, $CDCl_3$) of **S4**.

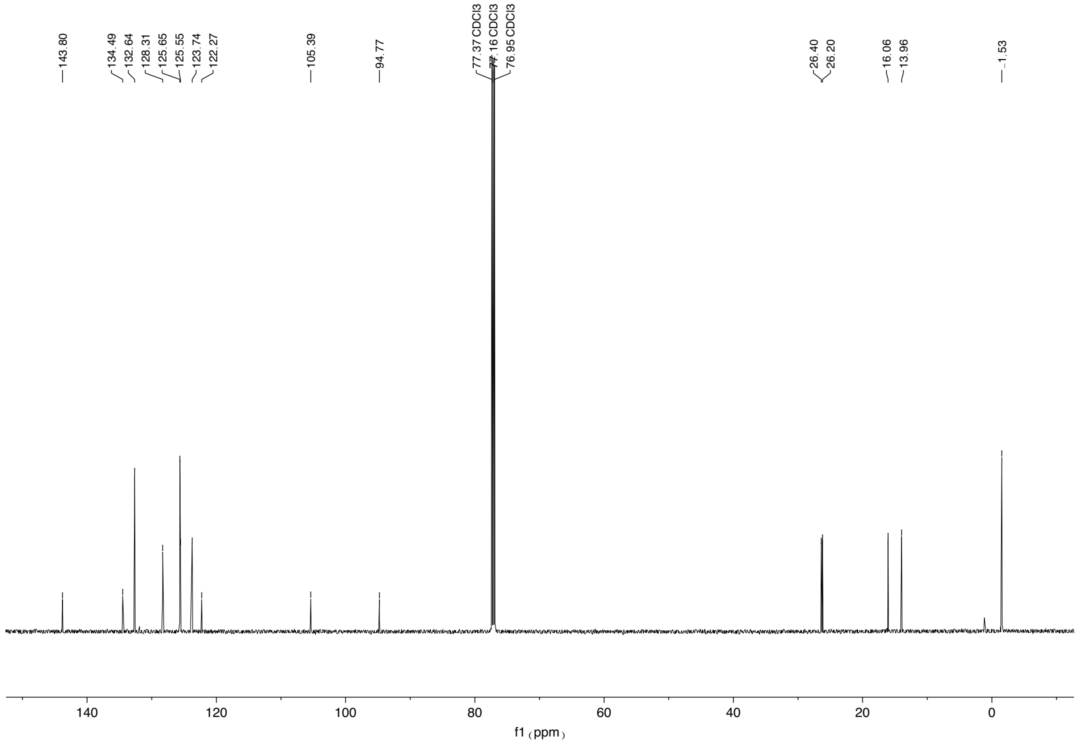

Figure S8. ^{1}H NMR Spectrum (400 MHz, C_6D_6) of **S5**.

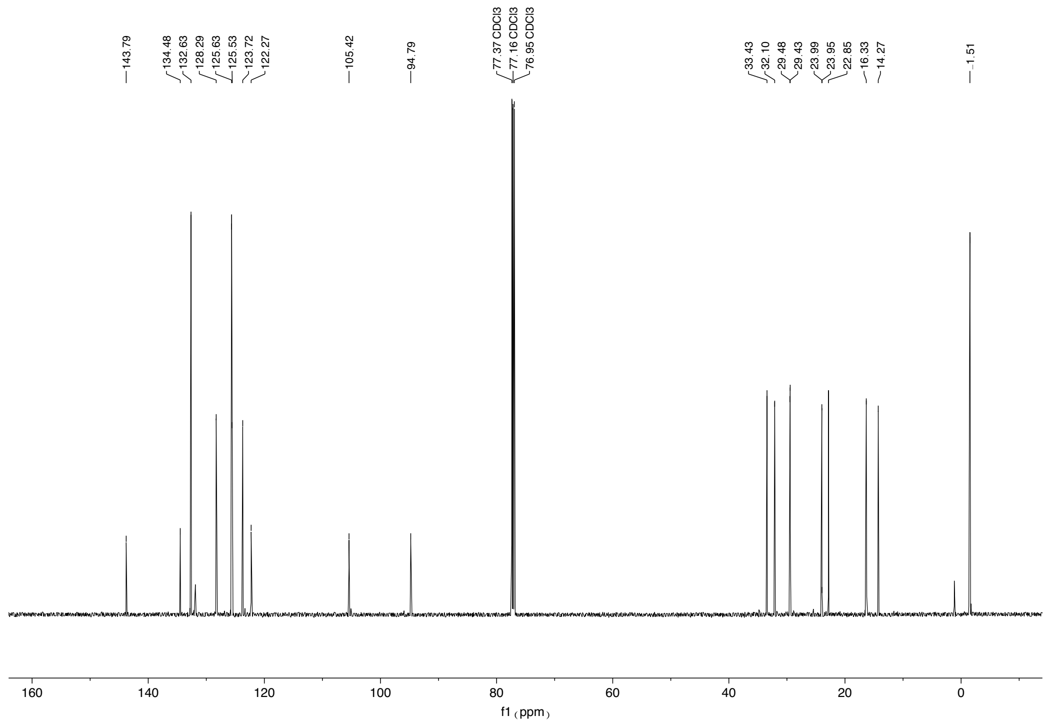

Figure S9. $^{13}C\{^1H\}$ NMR Spectrum (151 MHz, C_6D_6) of **S5**.

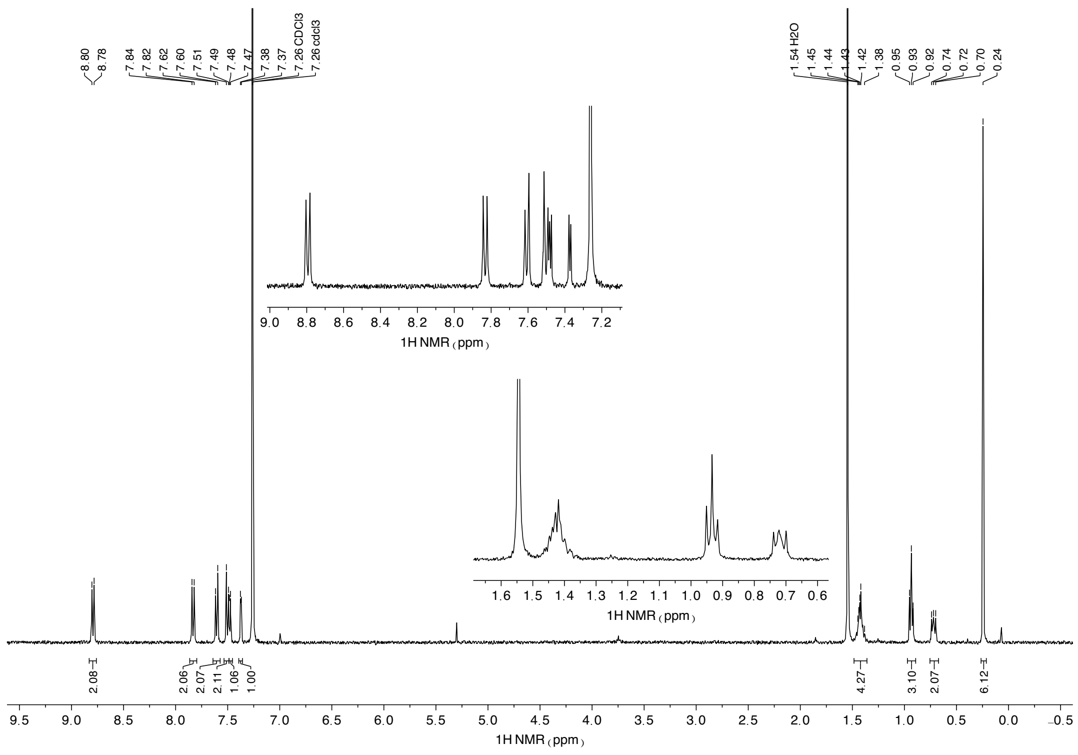

Figure S10. ^1H NMR Spectrum (400 MHz, C_6D_6) of **2-mon**.

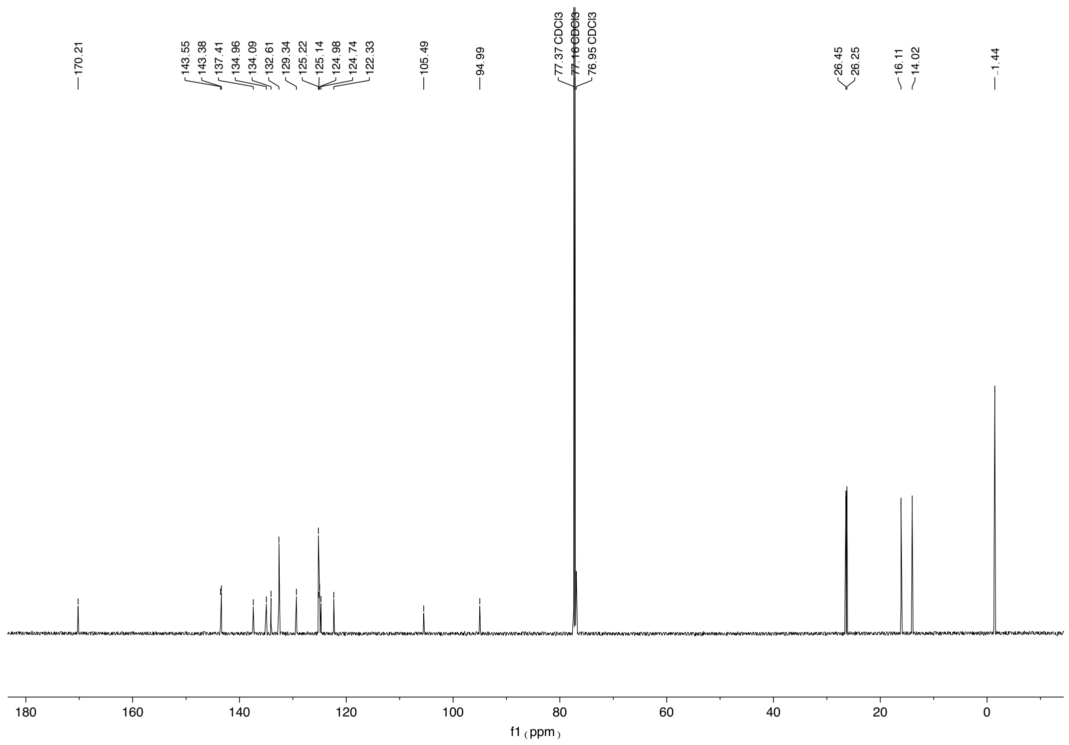

Figure S11. $^{13}C\{^{1}H\}$ NMR Spectrum (151 MHz, THF-d_8) of **2-mon**.

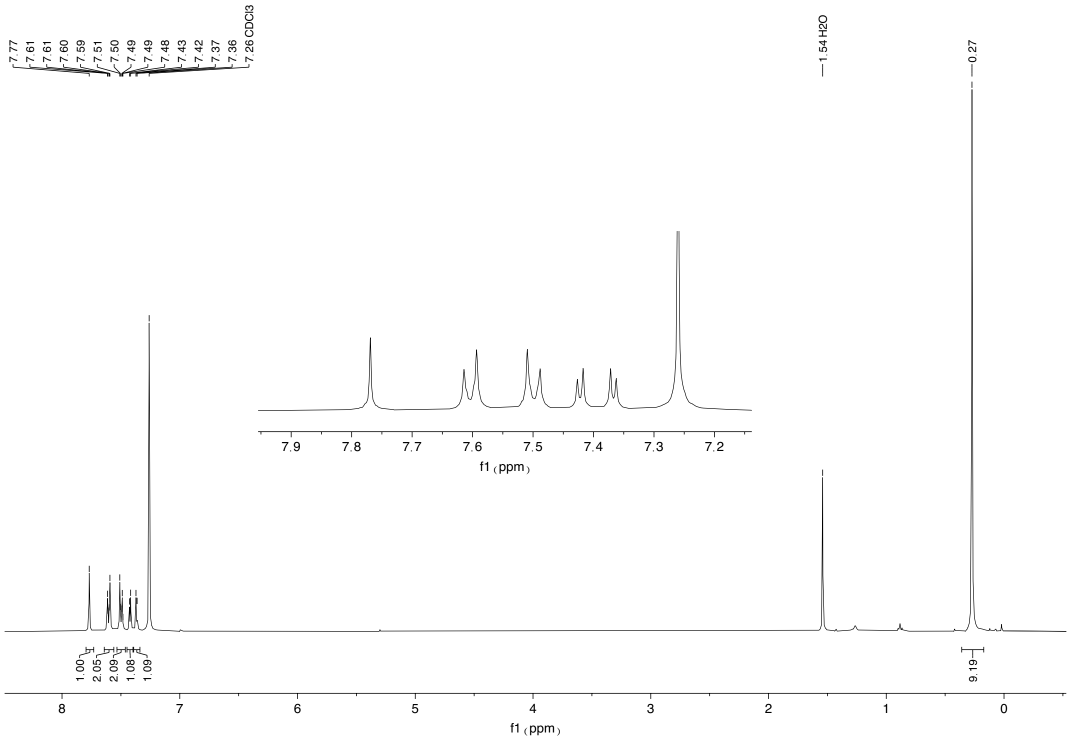

Figure S12. ^{1}H NMR Spectrum (400 MHz, C_6D_6) of **3-mon**.

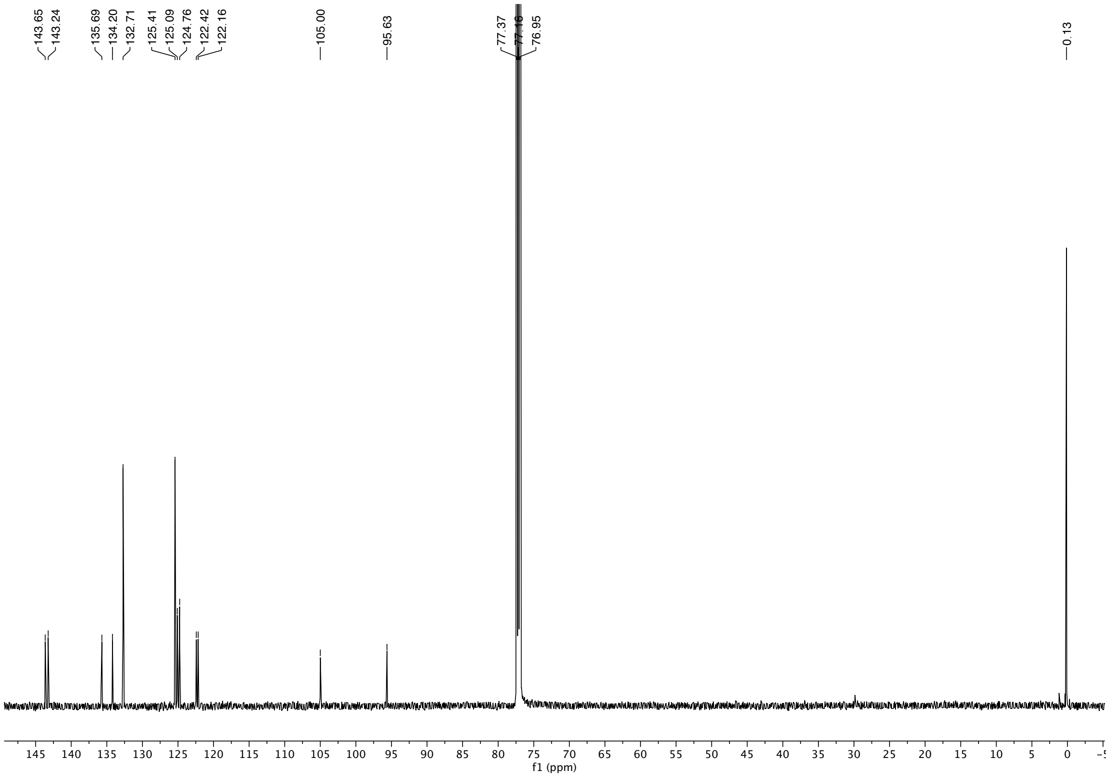

Figure S13. $^{13}C\{^{1}H\}$ NMR Spectrum (151 MHz, THF-d_8) of **3-mon**.

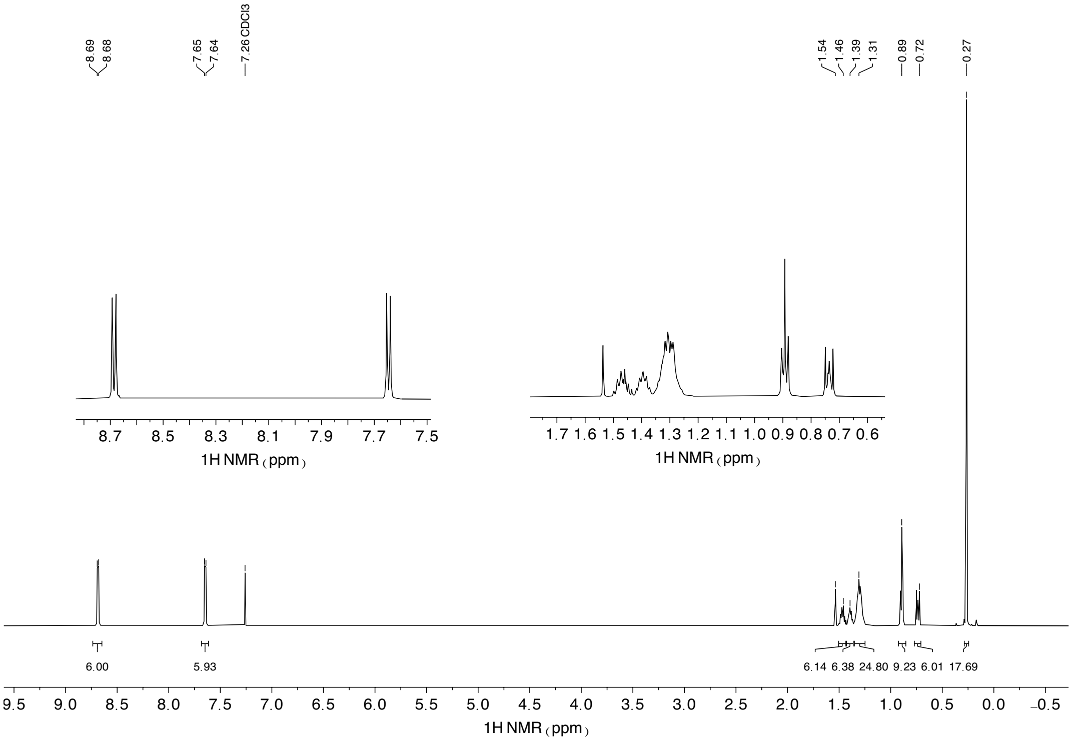

Figure S14. ^{1}H NMR Spectrum (600 MHz, C_6D_6) of **1-mon-oct**.

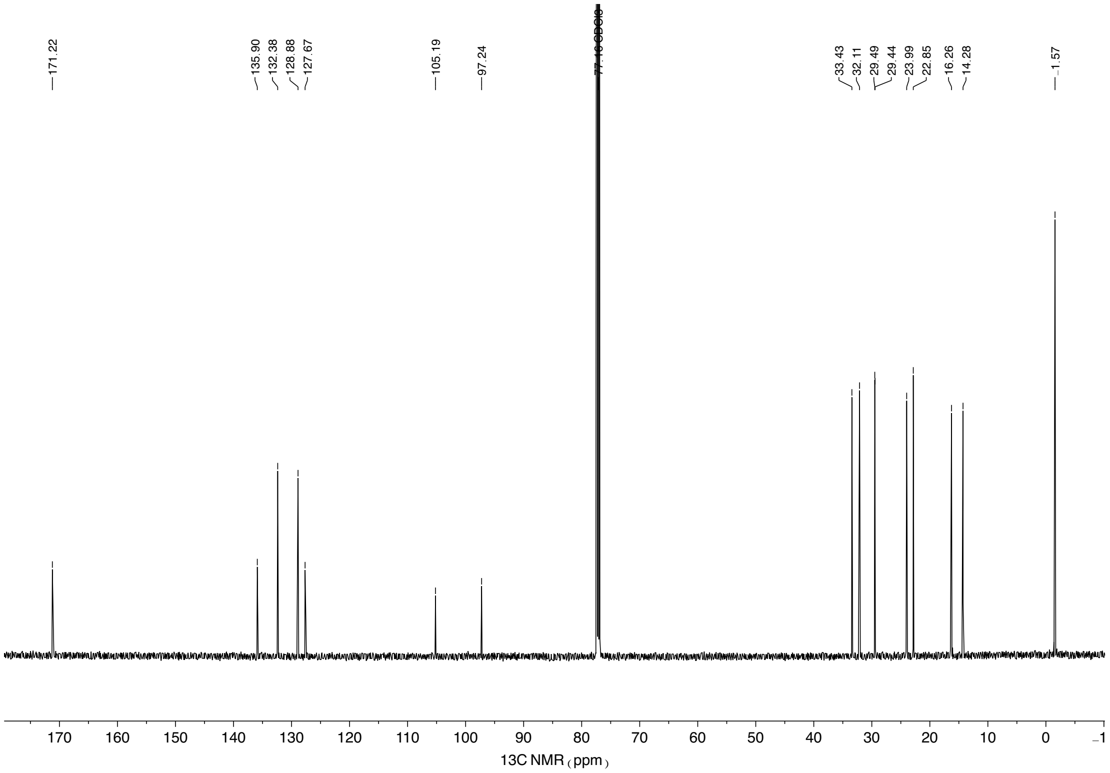

Figure S15. $^{13}C\{^{1}H\}$ NMR Spectrum (151 MHz, C_6D_6) of **1-mon-oct**.

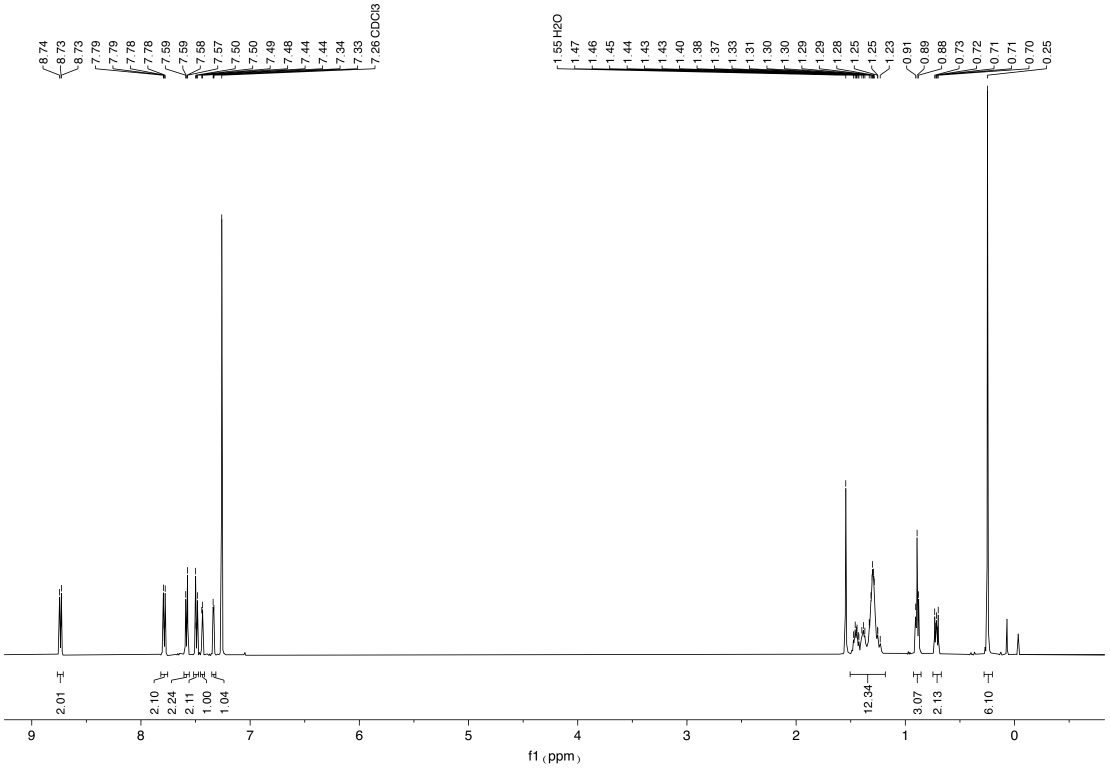

Figure S16. ^{1}H NMR Spectrum (600 MHz, C_6D_6) of **2-mon-oct**.

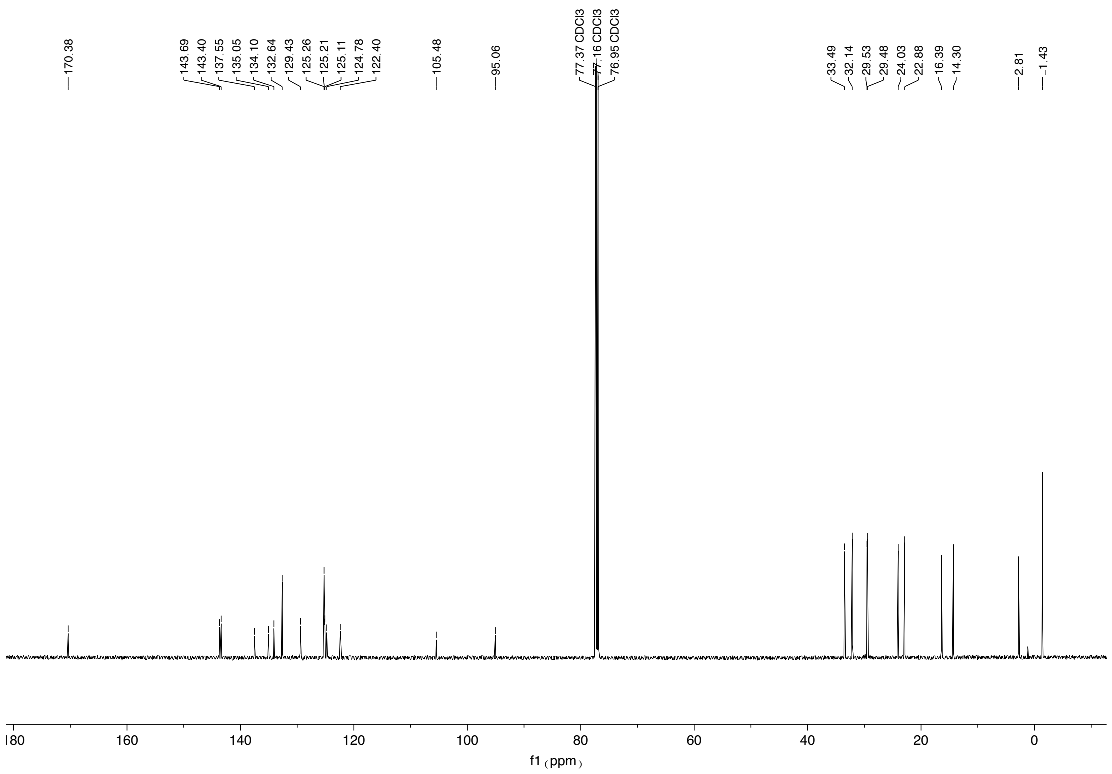

Figure S17. $^{13}C\{^{1}H\}$ NMR Spectrum (151 MHz, C_6D_6) of **2-mon-oct**.

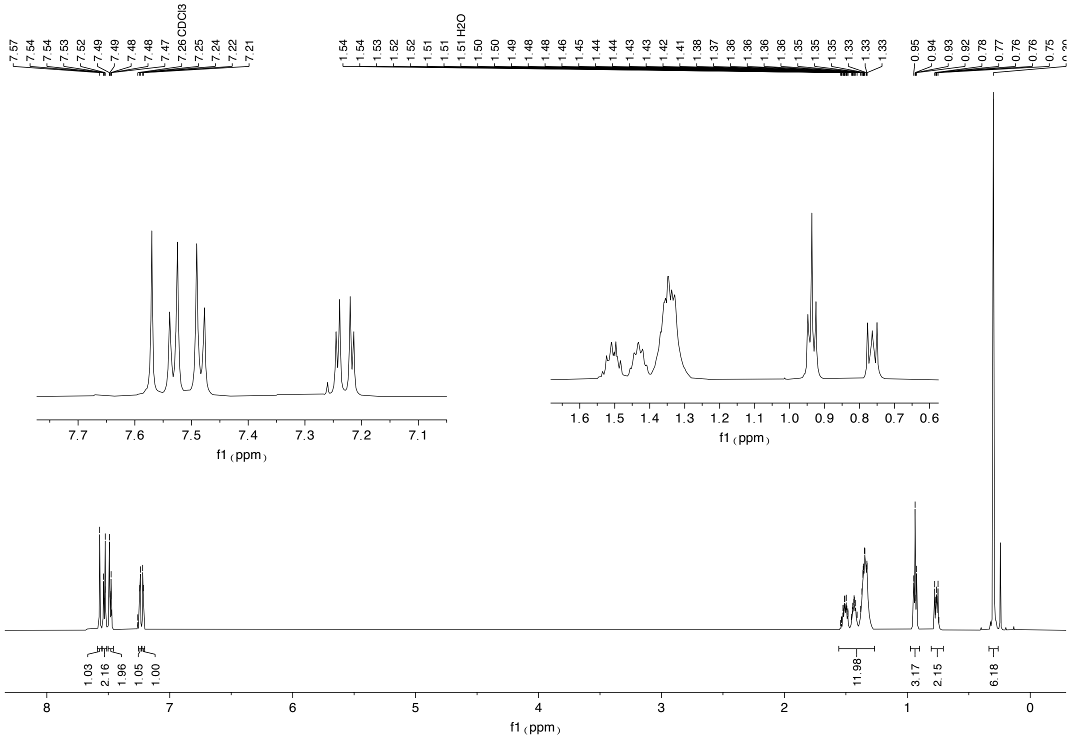

Figure S18. ^{1}H NMR Spectrum (600 MHz, C_6D_6) of **3-mon-oct**.

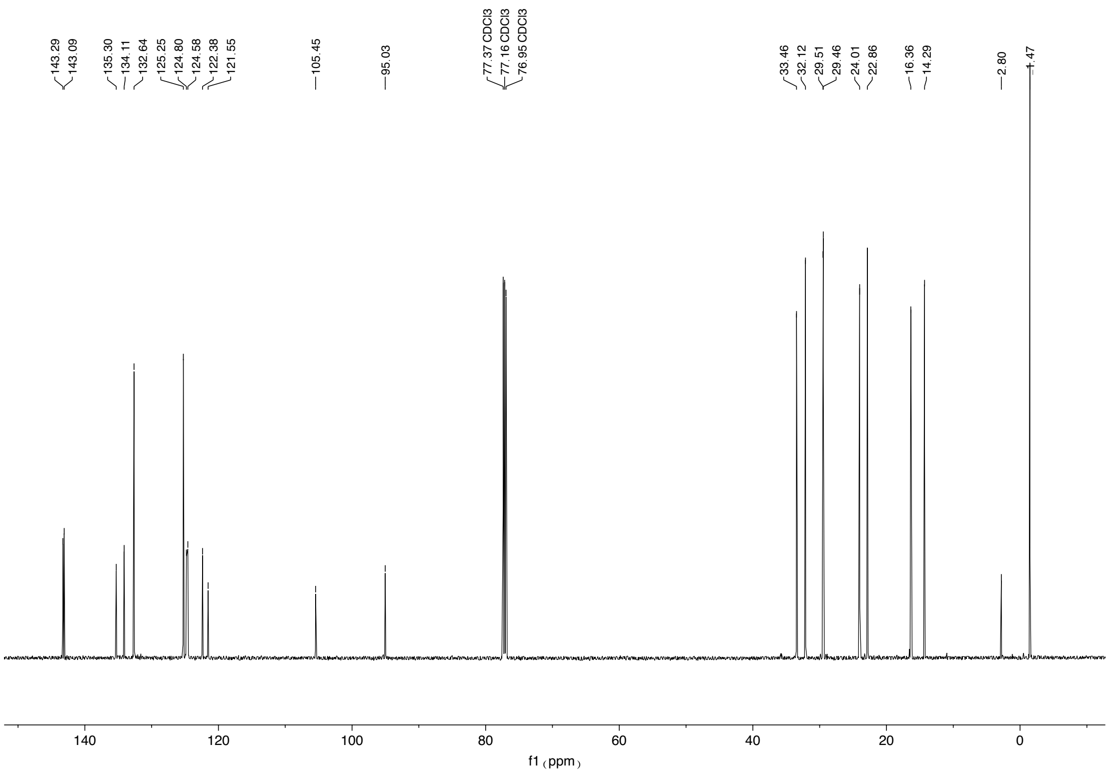

Figure S19. $^{13}C\{^{1}H\}$ NMR Spectrum (151 MHz, C_6D_6) of **3-mon-oct**.

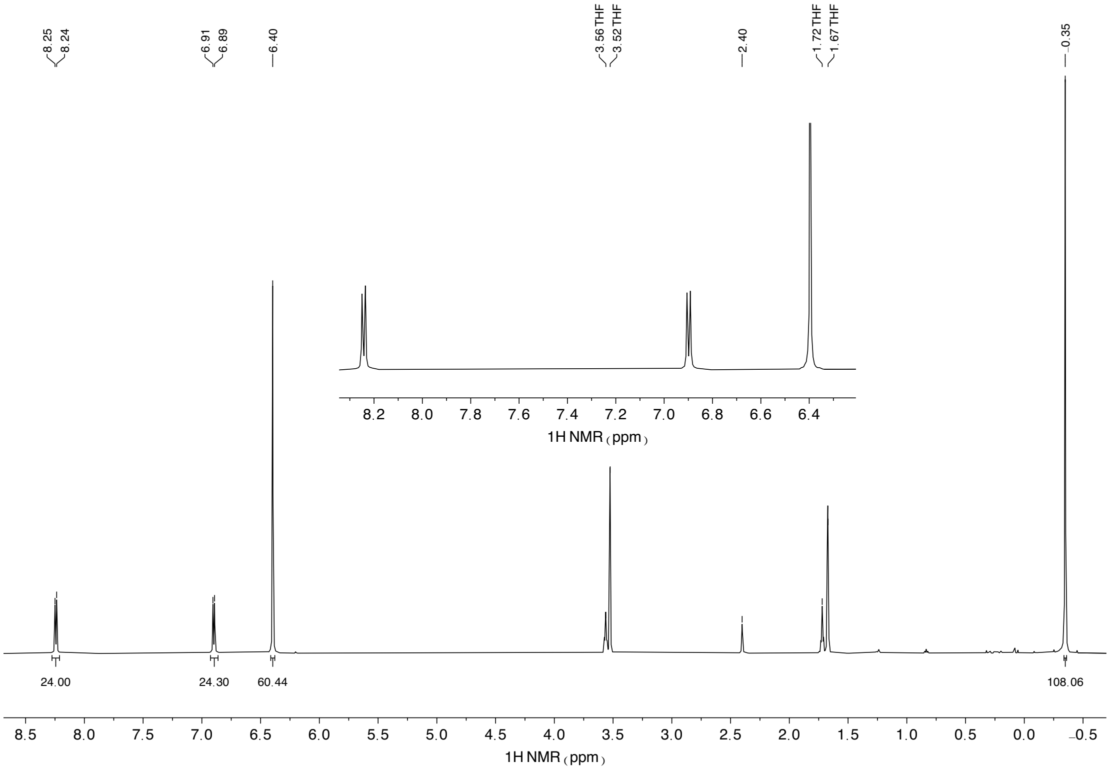

Figure S20. ^{1}H NMR Spectrum (600 MHz, C_6D_6) of **1-Zr**.

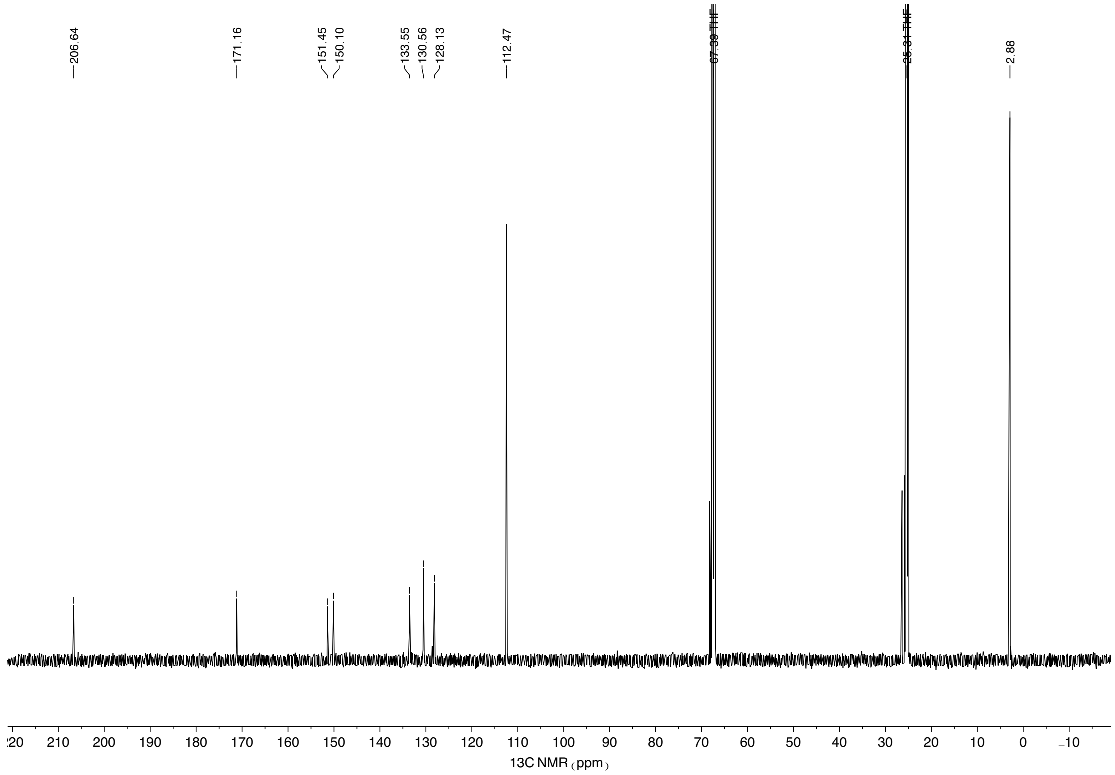

Figure S21. $^{13}C\{^{1}H\}$ NMR Spectrum (151 MHz, C_6D_6) of **1-Zr**.

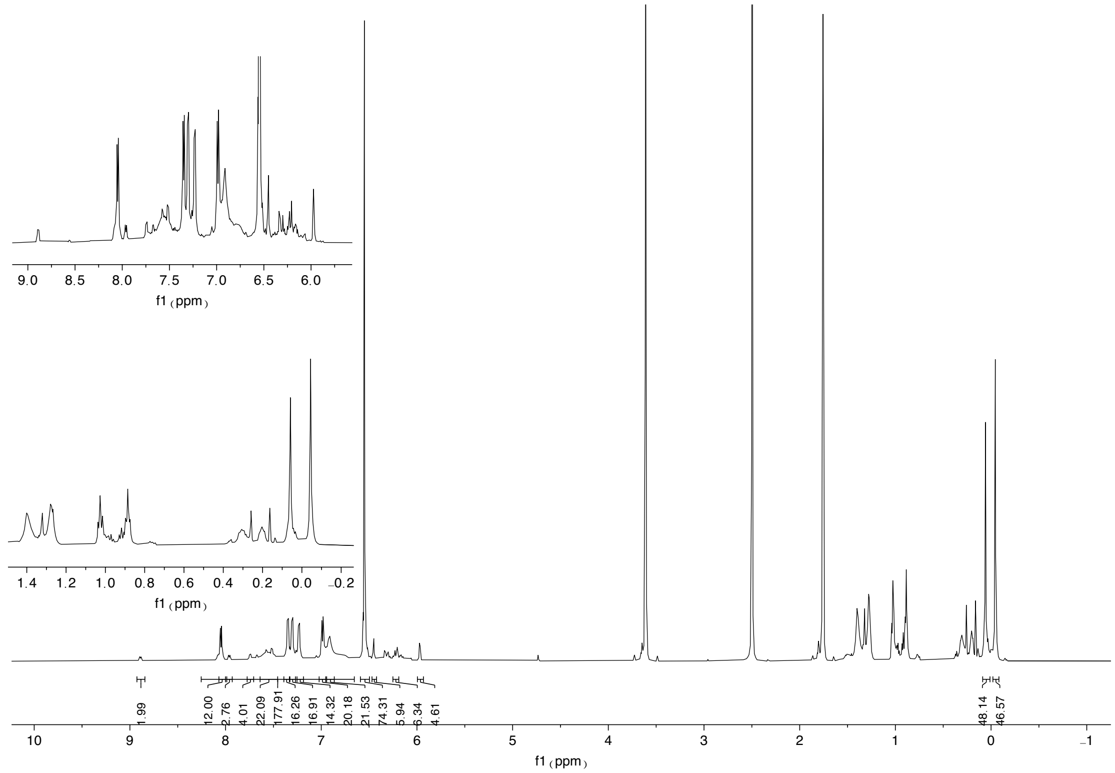

Figure S22. ^{1}H NMR Spectrum (600 MHz, C_6D_6) of **2-Zr**.

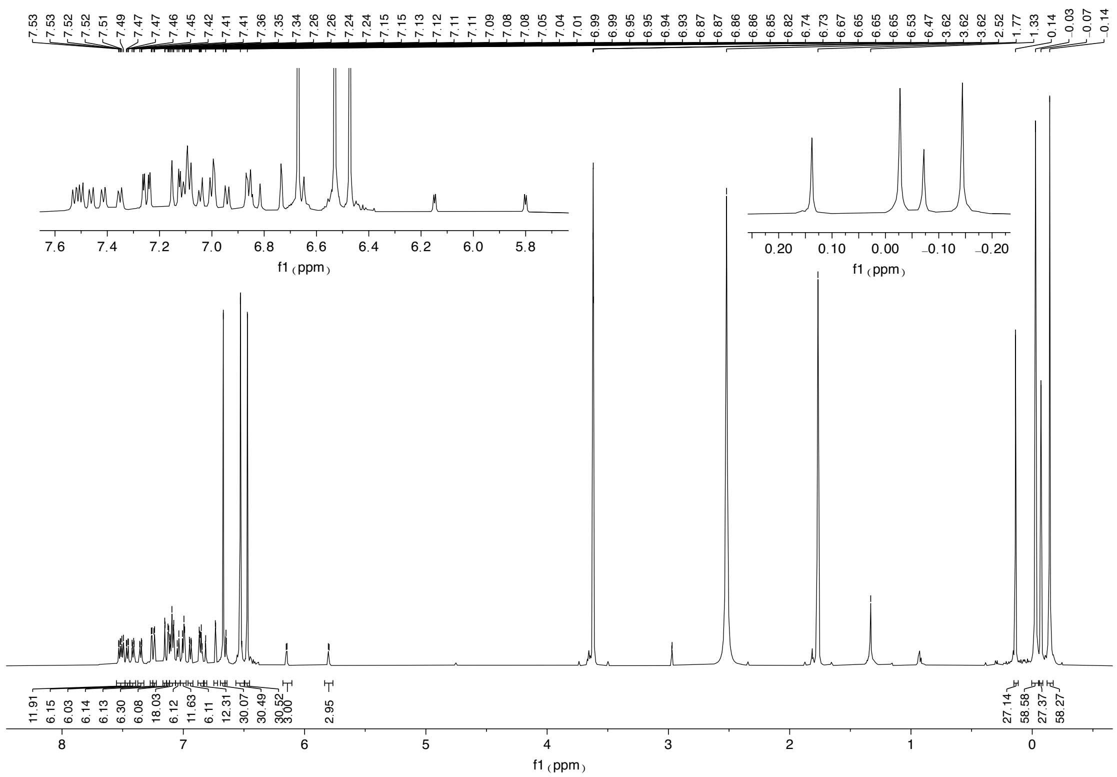

Figure S23. ^{1}H NMR Spectrum (600 MHz, C_6D_6) of **3-Zr**.

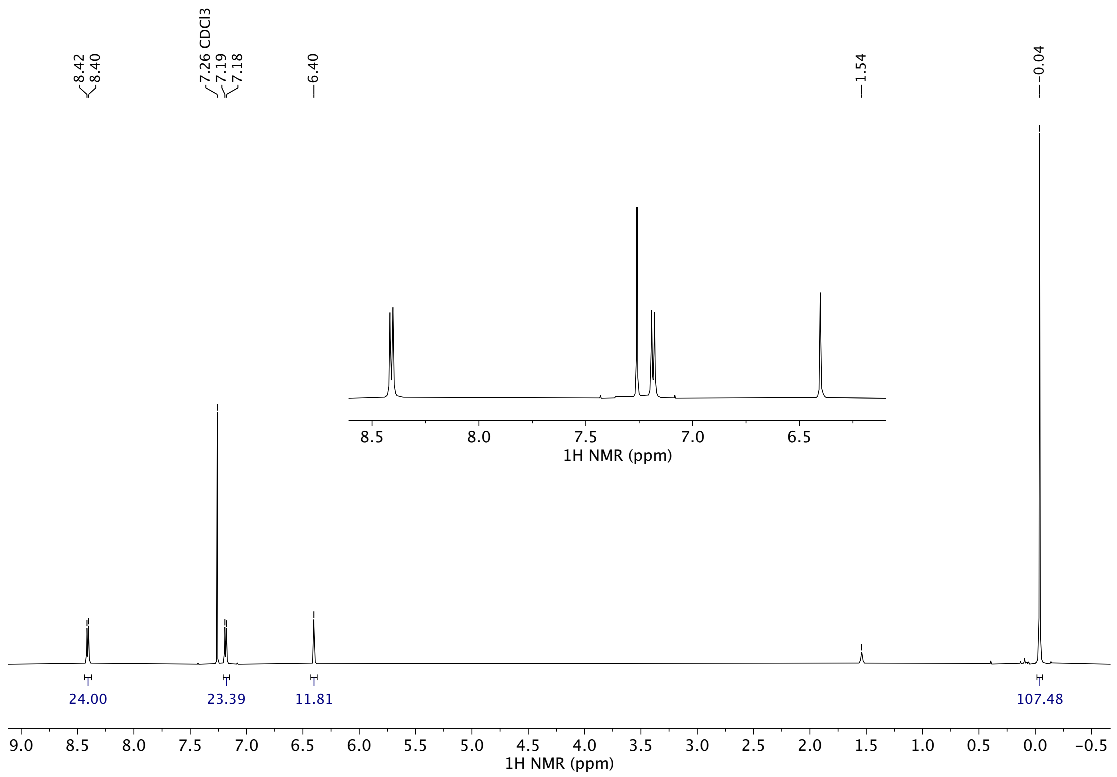

Figure S24. ^{1}H NMR Spectrum (600 MHz, C_6D_6) of **1**.

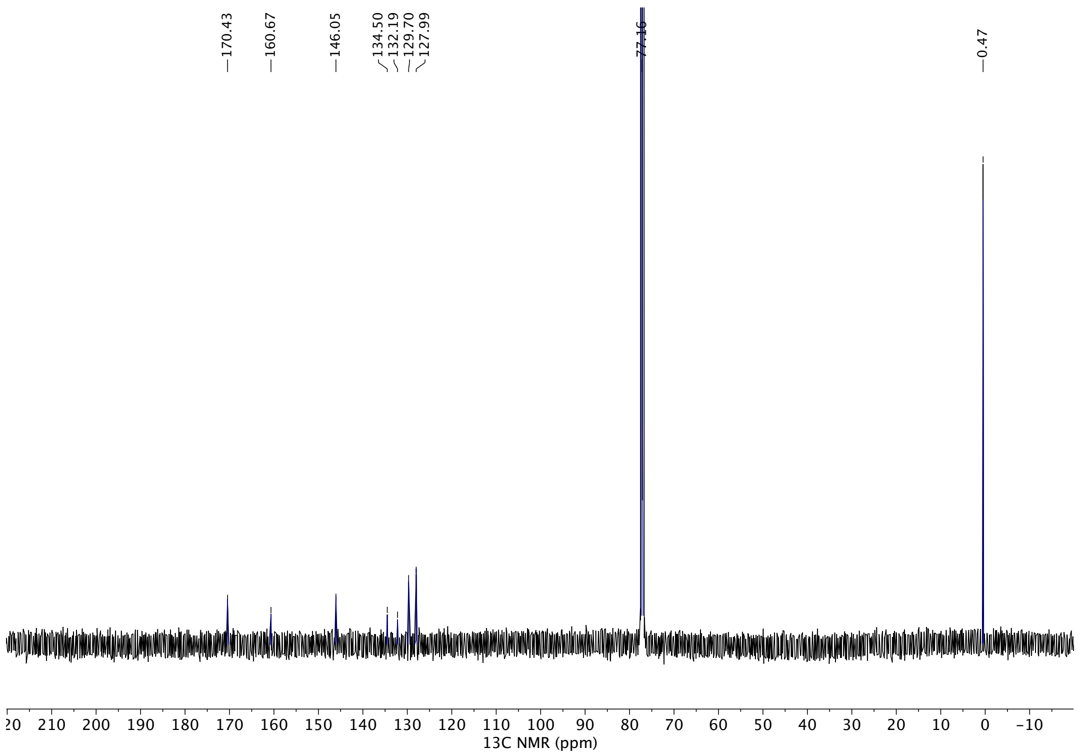

Figure S25. $^{13}C\{^{1}H\}$ NMR Spectrum (151 MHz, C_6D_6) of **1**.

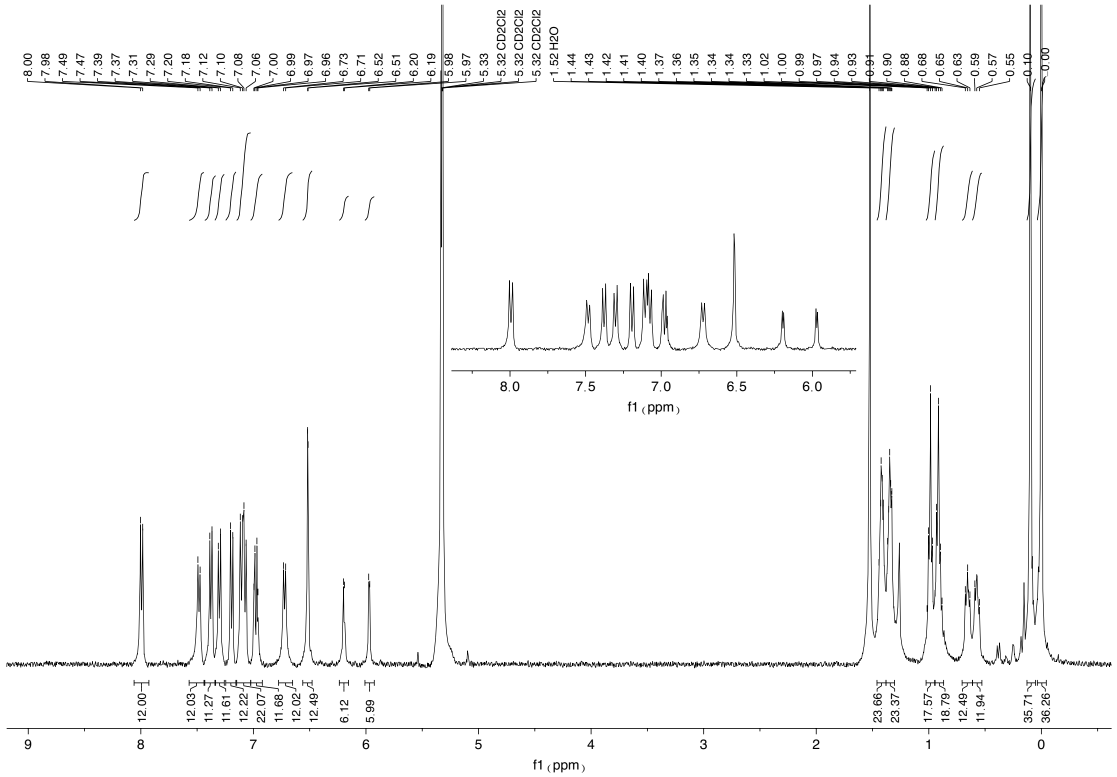

Figure S26. ^{1}H NMR Spectrum (600 MHz, C_6D_6) of **2**.

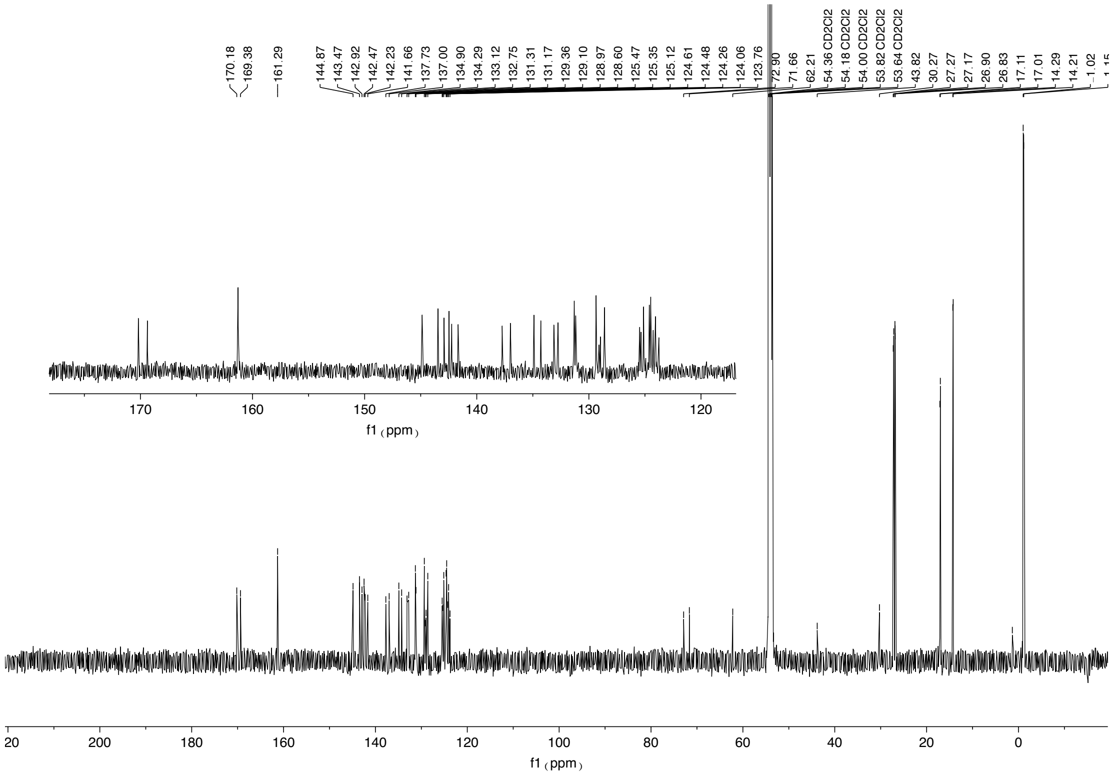

Figure S27. $^{13}C\{^{1}H\}$ NMR Spectrum (151 MHz, C_6D_6) of **2**.

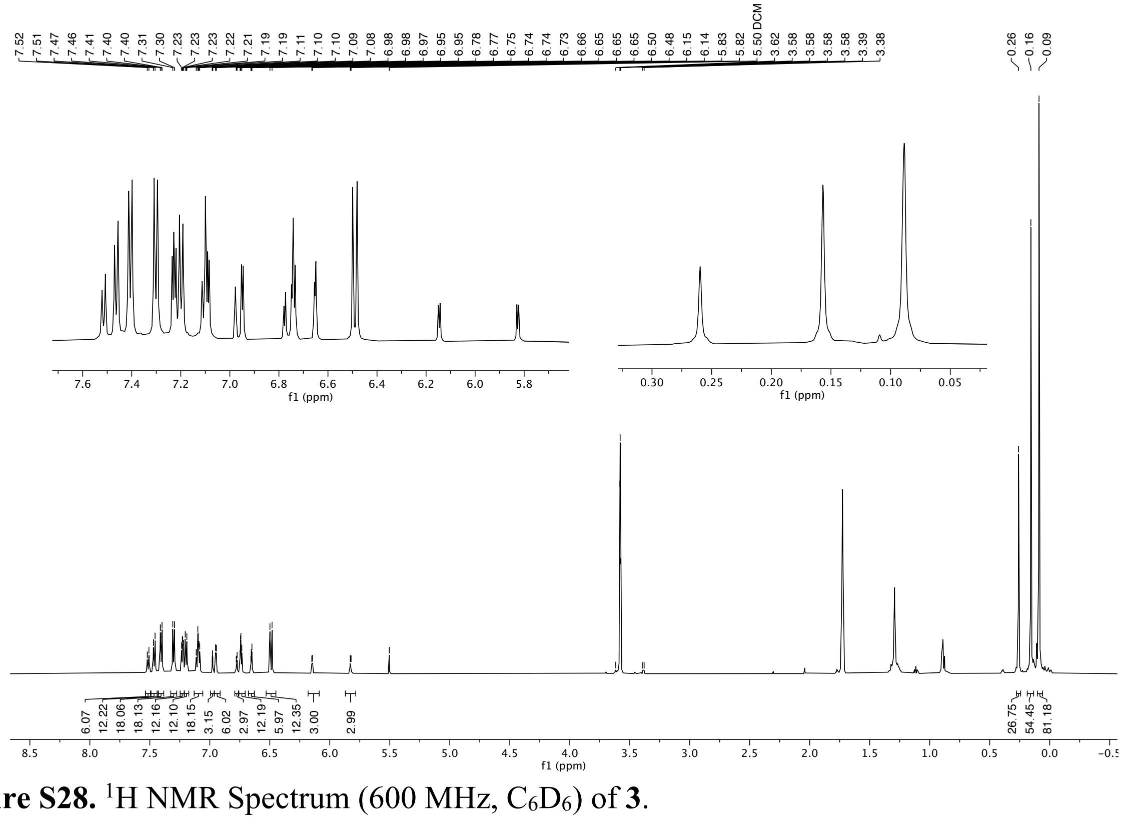

Figure S28. ^{1}H NMR Spectrum (600 MHz, C_6D_6) of **3**.

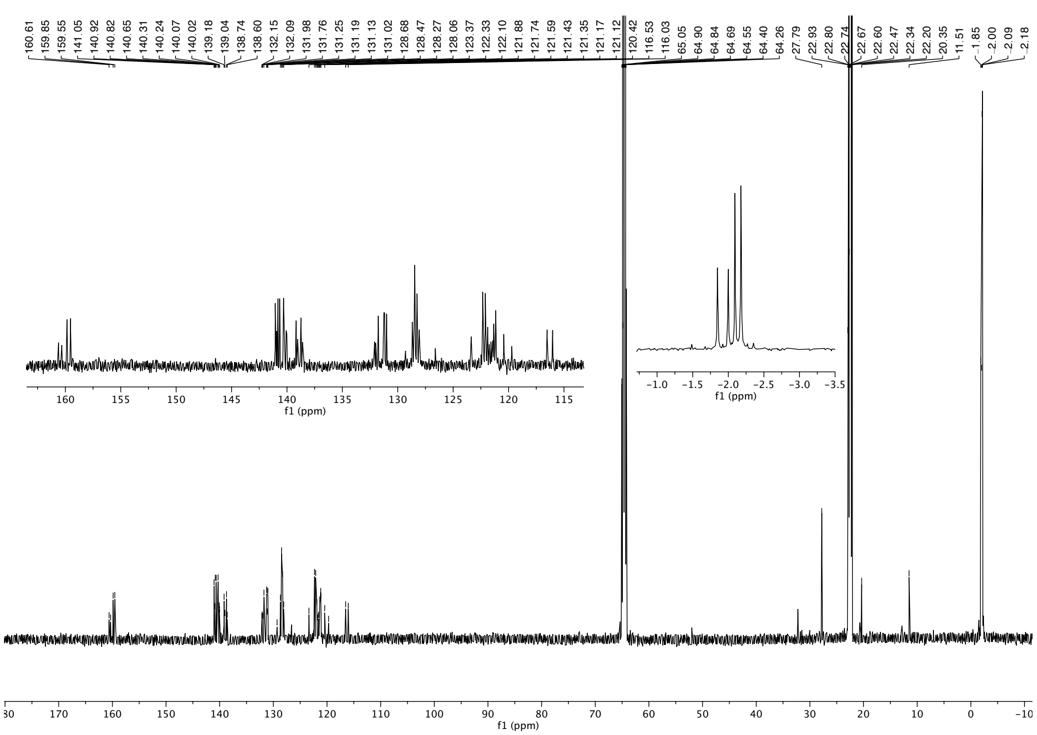

Figure S29. $^{13}C\{^{1}H\}$ NMR Spectrum (151 MHz, C_6D_6) of **3**.

MALDI-TOF Spectrometry

All MALDI-TOF spectrometry for **2** and **3** was conducted in positive mode using *trans*-2-[3-(4-*tert*-butylphenyl)-2-methyl-2-propenylidene]malononitrile (DCTB) as a matrix. The spectrum for **2** was acquired on an Applied Biosystems Voyager DE Pro in reflectron mode. **3** proved significantly more difficult to ionize, and only a low resolution spectrum in linear mode was achievable on the same instrument (Figure S31). The high resolution isotope pattern of **3** (Figure S31, inset) was acquired on a Bruker Autoflex Max in reflectron mode.

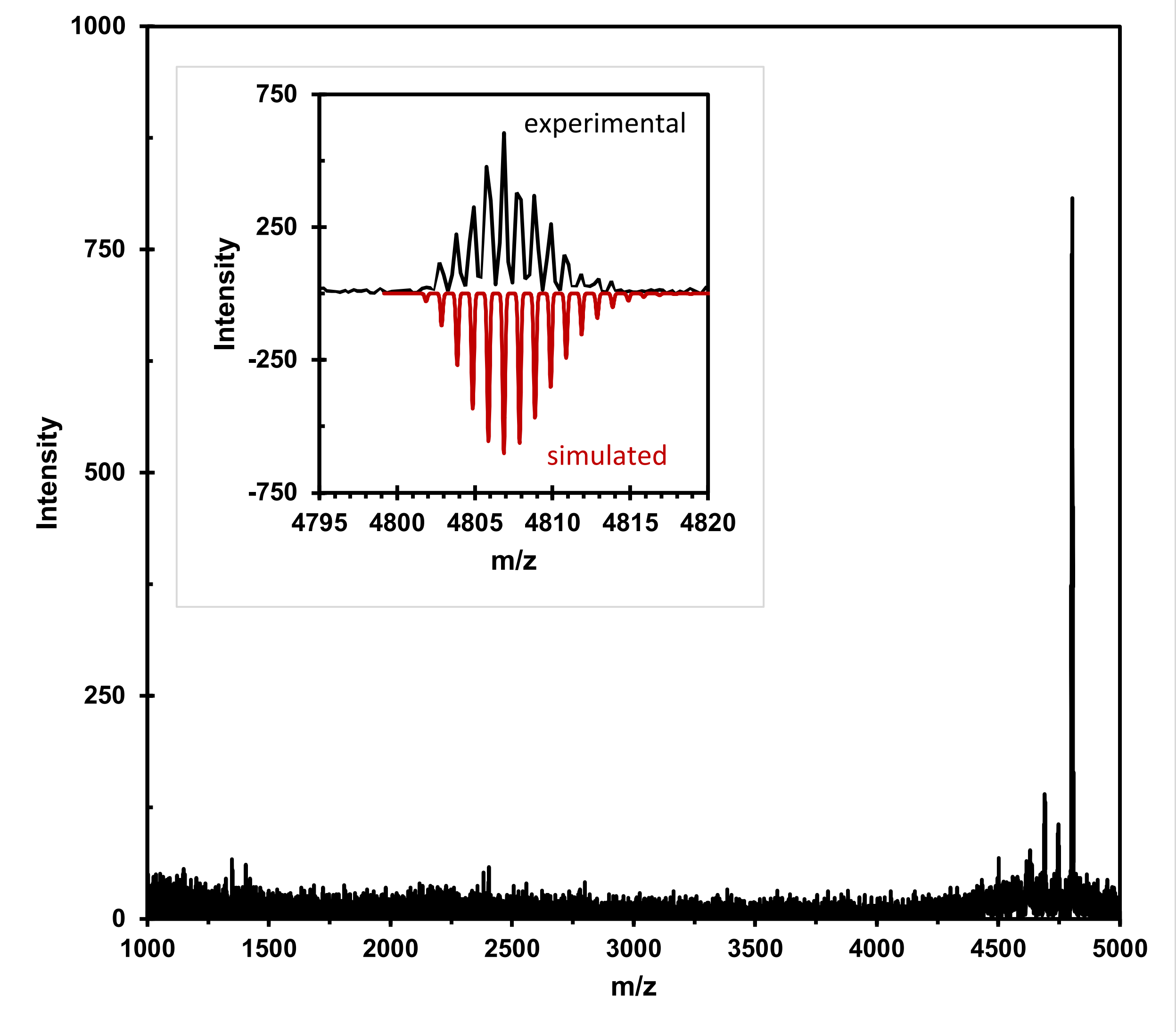

Figure S30. MALDI-TOF spectra of **2** from 1000-5000 amu. Inset depicts the experimental (black) and simulated (red) isotope pattern of **2**.

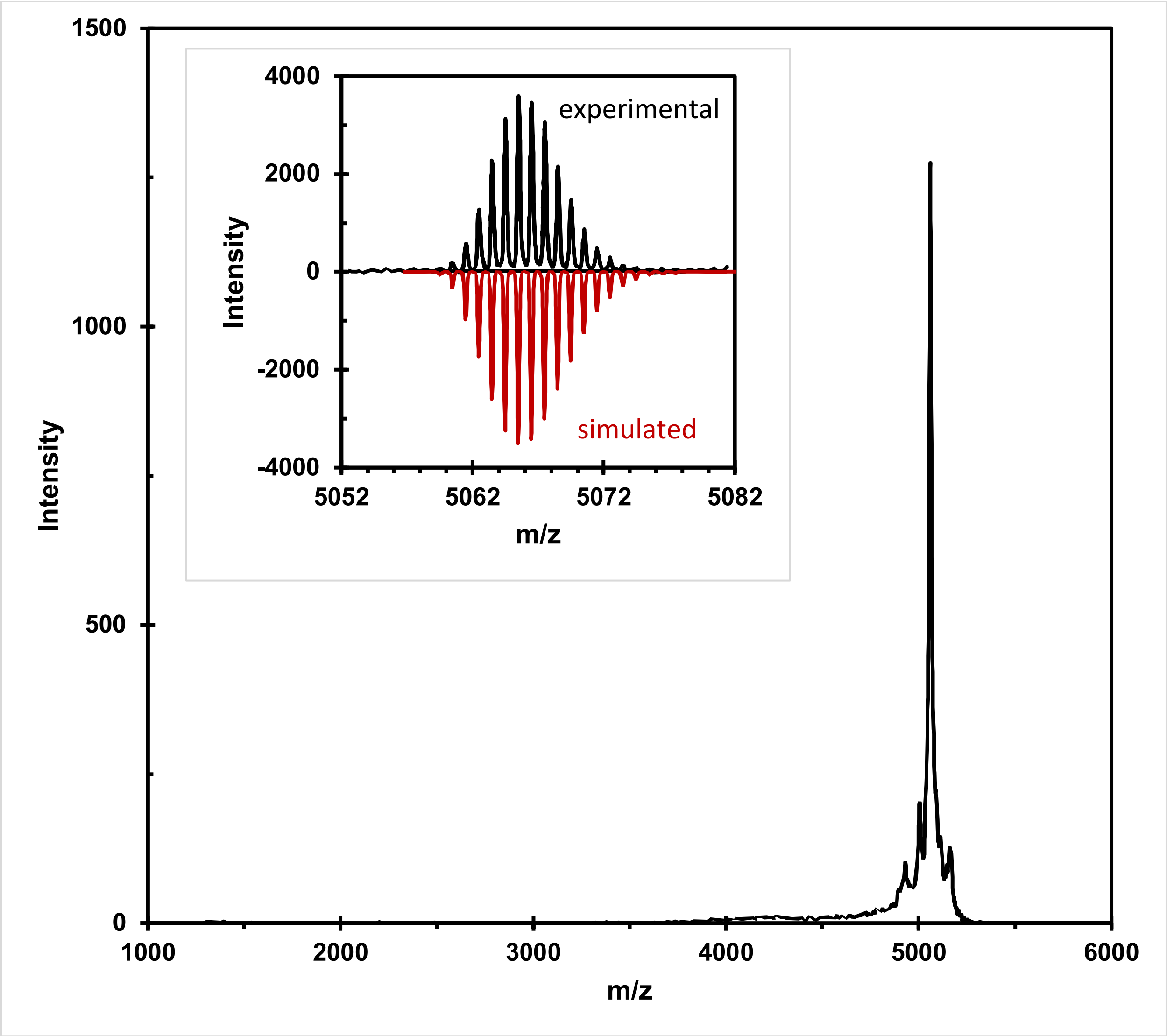

Figure S31. MALDI-TOF spectra of **3** from 1000-6000 amu. Inset depicts the experimental (black) and simulated (red) isotope pattern of **3**.

Absorption and Emission Spectroscopy

UV-Vis and fluorescence spectroscopies were performed on a Varian 5000 UV-Vis-NIR spectrometer and Nanolog Spectrofluorimeter respectively using quartz cuvettes with a path length of 1 cm. All UV-vis and fluorescence spectra were collected in DCM.

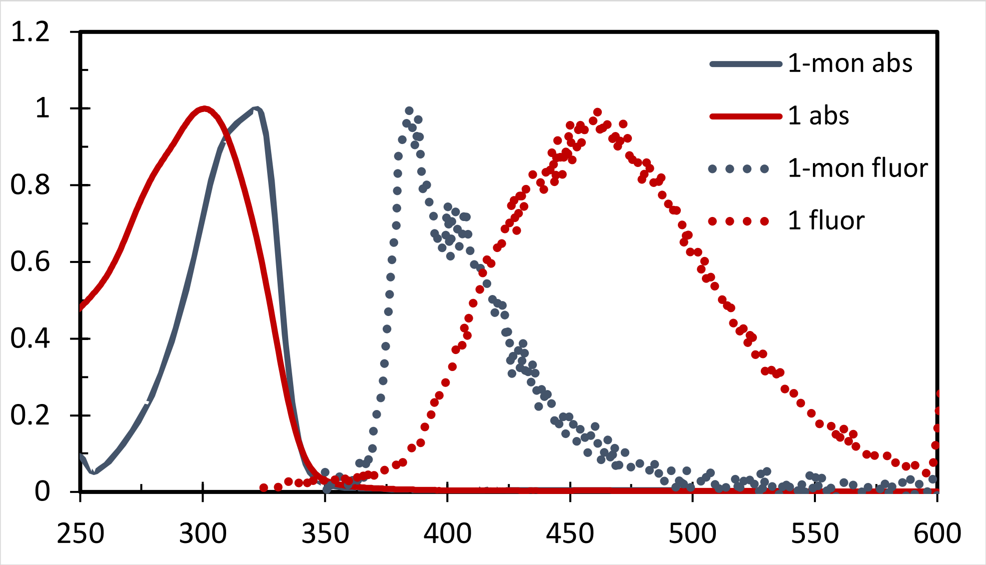

Figure S32. Normalized absorption (solid lines) and emission (dotted lines) for **1-mon** (blue) and **1** (red) in DCM at 6.00x10^{-6} M and 2.91x10^{-6} M respectively.

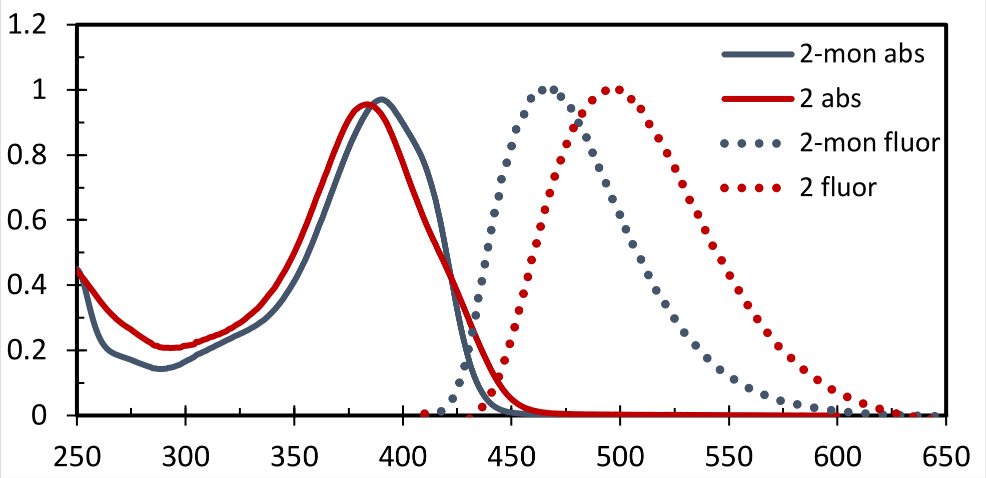

Figure S33. Normalized absorption (solid lines) and emission (dotted lines) for **2-mon** (blue) and **2** (red) in DCM at 3.17x10^{-5} M and 1.16x10^{-5} M respectively.

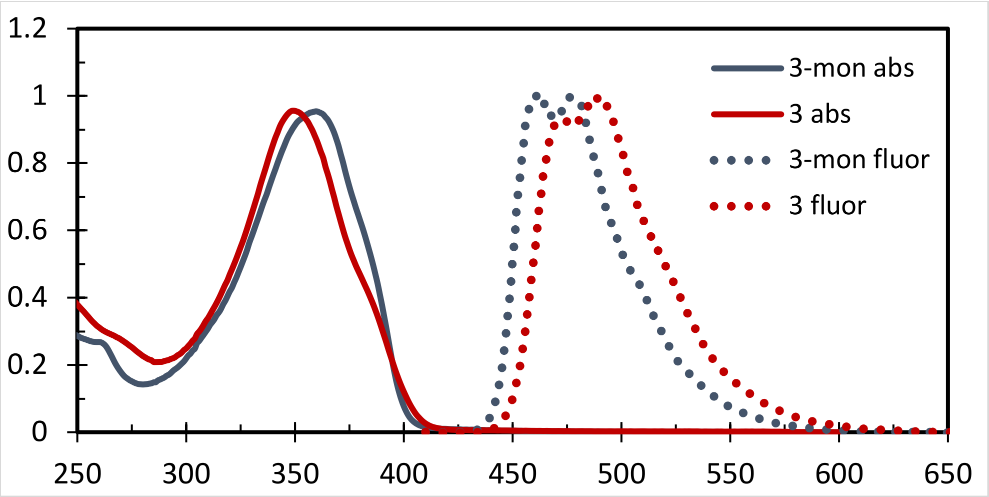

Figure S34. Normalized absorption (solid lines) and emission (dotted lines) for **3-mon** (blue) and **3** (red) in DCM at 4.04x10^{-5} M and 1.30x10^{-5} M respectively.

X-ray Crystallography

Synchrotron data for **1-Zr** was collected at beamline 12.2.1 of the Advanced Light Source at Lawrence Berkeley National Laboratory. Frames were collected on a shutterless PHOTON II detector using radiation with a wavelength of 0.7288 Å selected by a Si(111) monochromator and focused to 200 μm^2 with a toroidal mirror. Data collection, integration, scaling, and space group determination was performed with Bruker APEX3 (v. 2016.5-0) software. Synchrotron data for **2-Zr**, and **3-Zr** was collected at wavelengths of 0.97918 Å (**2-Zr**) and 1.50 Å (**3-Zr**) on beamline 24-ID-C at the Advanced Photon Source, Argonne National Laboratory, which is equipped with a single axis MD2 goniometer, X-ray diffractometer and a Dectris Eiger2 16M pixel array detector at a distance of 150 mm. This single axis goniometer limited our achievable value of $\sin(\theta_{max})/\lambda$ but did not affect the unambiguous determination of this structure. All diffraction data were processed using the XDS suite of programs.[15] All structures were solved ab initio using direct methods in SHELXT.[16-17] Olex2 software was used for the refinement.[18]

Crystals of **1-Zr** were found to be uniformly weakly diffracting, exhibiting little diffraction beyond 0.90 Å. Positional disorder is present in two of the zirconocene "vertices" of the molecule; this was modeled with use of the AFIX 55 constraint and fixed 50% occupancies of the two disordered components. Additionally, these vertex units displayed varying extents of disorder within the *bis*-trimethylsilylzirconacyclopentadienyl moieties; this disorder was modeled in two positions using fixed 50% occupancies. To maintain reasonable anisotropic displacement parameters within the disordered moieties, RIGU, EADP, and SIMU constraints / restraints were used. The asymmetric unit of **1-Zr** contains 5.5 molecules of benzene-d_6 co-solvent which would be discreetly modeled; an additional 0.67 molecules of benzene-d_6 were modeled using a solvent mask. Hydrogen atoms were added using a riding model and refined isotropically. Attempts to refine hydrogen atom positions on atom C15 (part of a rotationally disordered trimethylsilyl group) using the appropriate

HFIX command did not lead to a converged refinement; thus, no hydrogen atoms have been placed on atom C15.

For **2-Zr** and **3-Zr**, the non-hydrogen atoms were located in successive difference Fourier syntheses and refined with anisotropic thermal parameters. All the hydrogen atoms were placed at the calculated positions and refined using a riding model with appropriate HFIX command. DISP instructions were used for each scattering factor types. In case of **2-Zr**, the data quality was very poor. There were trapped solvent molecule which could not be modelled and were squeezed out from the refinement model using solvent mask in Olex2. The data quality for **3-Zr** was much better, and most of the solvent benzene molecules in the structure could be modelled. The remaining disordered solvent molecules were squeezed out. Rigid bond restraints such as DELU, SIMU along with DFIX, DANG were used to stabilize the structure in both the cases especially in the case of hanging butyl chain in **2-Zr**.

Identification code	**1-Zr**
Empirical formula	$C_{241.02}D_{37.02}H_{213}N_{12}Si_{12}Zr_6$
Formula weight	4236.42
Temperature/K	273.15
Crystal system	monoclinic
Space group	*C*2/*c*
a/Å	63.908(6)
b/Å	23.962(2)
c/Å	35.276(3)
α/°	90
β/°	94.193(4)
γ/°	90
Volume/Å^3	53876(9)
Z	8
μ/mm^{-1}	0.344
F(000)	17505.0
Crystal size/mm^3	0.11 × 0.1 × 0.06
Radiation	synchrotron (λ = 0.7288)
2Θ range for data collection/°	2.182 to 48.004
Index ranges	$-71 \leq h \leq 71$, $-26 \leq k \leq 26$, $-39 \leq l \leq 39$
Reflections collected	243662
Independent reflections	38943 [R_{int} = 0.0512, R_{sigma} = 0.0352]
Data/restraints/parameters	38943/445/2626
Goodness-of-fit on F^2	1.423
Final R indexes [I>=2σ (I)]	R_1 = 0.0875,

	wR_2 = 0.3001
Final R indexes [all data]	R_1 = 0.1028, wR_2 = 0.3176
Largest diff. peak/hole / *e* Å^{-3}	1.09/–1.84

Table S7. Crystal data and structure refinement for **1-Zr**.

Identification code	**2-Zr**
Empirical formula	C_{360} H_{372} N_{12} S_{12} Si_{12} Zr_6 [+ solvent]
Formula weight	6135.84
Temperature/K	100
Crystal system	Monoclinic
Space group	$P2_1/c$
a/Å	13.820(3)
b/Å	46.380(9)
c/Å	69.940(14)
α/°	90
β/°	93.745(19)
γ/°	90
Volume/Å^3	44734 (16)
Z	4
ρ_{calc}g/cm^3	0.911
μ/mm^{-1}	0.622
F(000)	12864
Crystal size/mm^3	0.210×0.190×0.015
Radiation	Synchrotron (λ = 0.97918)
2Θ range for data collection/°	0.73 to 25.19
Index ranges	$-11 \leq h \leq 10$, $-37 \leq k \leq 39$, $-58 \leq l \leq 53$
Reflections collected	77563
Independent reflections	27515
Data/restraints/parameters	27515/5861/2797
Goodness-of-fit on F^2	1.369
Final R indexes [I>=2σ (I)]	R_1 = 0.1393, wR_2 = 0.3894
Final R indexes [all data]	R_1 = 0.1726, wR_2 = 0.4169
Largest diff. peak/hole / e Å^{-3}	0.63/-0.43

Table S8. Crystal data and structure refinement for **2-Zr**.

Identification code	**3-Zr**
Empirical formula	C_{942} H_{906} S_{36} Si_{36} Zr_{18} [+ solvent]
Formula weight	16033.79
Temperature/K	100
Crystal system	trigonal

Space group	$P3_1$
a/Å	41.640(8)
b/Å	41.640(8)
c/Å	58.480(12)
α/°	90.00(3)
β/°	90.00(3)
γ/°	120.00(3)
Volume/Å^3	87813 (38)
Z	3
ρ_{calc}g/cm^3	0.910
μ/mm^{-1}	2.329
F(000)	25074
Crystal size/mm^3	0.195×0.050×0.010
Radiation	Synchrotron (λ = 1.5000)
2Θ range for data collection/°	1.40 to 52.22
Index ranges	$-42 \le h \le 42$, $-43 \le k \le 43$, $-61 \le l \le 61$
Reflections collected	697469
Independent reflections	142827
Data/restraints/parameters	142827/12548/7526
Goodness-of-fit on F^2	0.997
Final R indexes [I>=2σ (I)]	R_1 = 0.0810, wR_2 = 0.2227
Final R indexes [all data]	R_1 = 0.0875, wR_2 = 0.2318
Largest diff. peak/hole / e Å^{-3}	1.06/-0.37
Flack parameter	0.433 (7)

Table S9. Crystal data and structure refinement for **3-Zr**.

References

1) Marques-Gonzalez, S.; Parthey, M.; Yufit, D. S.; Howard, J. A. K.; Kaupp, M.; Low, P. J. Combined Spectroscopic and Quantum Chemical Study of [trans-Ru(C≡CC6H4R1-4)2(dppe)2]n+ and [trans-Ru(C≡CC6H4R1-4)(C≡CC6H4R2-4)(dppe)2]n+ (n = 0, 1) Complexes: Interpretations beyond the Lowest Energy Conformer Paradigm. *Organometallics,* **2014**, *33*, 4947–4963
2) Bao, H.; Zhou, B.; Luo, S. P.; Xu, Z.; Jin, H.; Liu, Y. P/N Heteroleptic Cu(I)-Photosensitizer-Catalyzed Deoxygenative Radical Alkylation of Aromatic Alkynes with Alkyl Aldehydes Using Dipropylamine as a Traceless Linker Agent. *ACS Catal.* **2020**, *10*, 7563–7572.
3) Kulkarni, R.; Huang, J.; Trunk, M.; Burmeister, D.; Amsalem, P.; Müller, J.; Martin, A.; Koch, N.; Kassa, D.; Bojdys, M. J. Direct growth of crystalline triazine-based graphdiyne using surface-assisted deprotection–polymerization. *Chem. Sci.*, *2021*, *12*, 12661-12666.
4) Nitschke, J. R.; Zu, S.; Tilley, T. D. New Zirconocene-Coupling Route to Large, Functionalized Macrocycles. *J. Am. Chem. Soc.,* **2000***, 122, 10345–10352.*
5) Zhang, W.; Moore, J. S. Arylene Ethynylene Macrocycles Prepared by Precipitation-Driven Alkyne Metathesis. *J. Am. Chem. Soc.* **2004**, *126*, 40, 12796.

6) Hasell, T.; Wu, X.; Jones, J. T. A.; Basca, J.; Steiner, A.; Mitra, T.; Trewin, A.; Adams, D. J.; Cooper, A. I. Triply Interlocked Covalent Organic Cages. *Nat. Chem.* **2010**, *2*, 750-755.
7) Li, F.; Clegg. J. K.; Lindoy, L. F.; Macquart, R. B.; Meehan, G. V. Metallosupramolecular self-assembly of a universal 3-ravel. *Nat. Commun.* **2011**, *2*, 205.
8) Gessner, V. H., Tannaci, J. F., Miller, A. D. & Tilley, T. D. Assembly of Macrocycles by Zirconocene-Mediated, Reversible Carbon−Carbon Bond Formation. *Acc. Chem. Res.* **44**, 435–446 (2011).
9) Zhang, J.; Moore, J. S. Nanoarchitectures. 3. Aggregation of hexa(phenylacetylene) macrocycles in solution: a model system for studying .pi.-.pi. interactions. *J. Am. Chem. Soc.* **1994**, *116*, 4227–4239.
10) Zhang, J.; Pesak, D. J.; Ludwick, J. L.; Moore, J. S. Geometrically-Controlled and Site-Specifically-Functionalized Phenylacetylene Macrocycles. *J. Am. Chem. Soc.* **1992**, *114*, 9701–9702.
11) Au-Yeung, H. Y.; Deng, Y. Distinctive features and challenges in catenane chemistry. *Chem. Sci.*, **2022**, *13*, 3315-3334.
12) Castle, T.; Evans, M. E.; Hyde, S. T. Ravels: Knot-Free but not Free. Novel Entanglements of Graphs in 3-space. *New J. Chem.***,** **2008**, *32*, 1484-1492.
13) Hyde, S. T.; Friedrichs, O. D. From Untangled Graphs and Nets to Tangled Materials. **2011**, *13*, 676–683.
14) Castle, T.; Evans, M. E.; Hyde, S. T. Entanglement of Embedded Graphs. *Prog. Theor. Phys. Suppl.* **2008**, *191*, 235–244
15) Kabsch, W. Integration, scaling, space-group assignment and post-refinement. *Acta Crystallogr. D Biol. Crystallogr.* **2010**, *66*, 133–144.
16) Sheldrick, G. M. *Acta Crystallogr.* **2015**, *A71*, 3–8.
17) Sheldrick, G. M. *Acta Crystallogr.* **2015**, *C71*, 3–8.
18) Dolomanov, O. V.; Bourhis, L. J.; Gildea, R. J.; Howard, J. A. K.; Puschmann. OLEX2: a Complete Structure Solution, Refinement and Analysis Program. *J. Appl. Crystallogr.* **2009**, *42*, 339–341.
